# Supplementary material for: Acceptability of surgical care in Uganda: a qualitative study on users and providers
Source: BMJ Open. 2023 Jul 31;13(7):e070479. doi: 10.1136/bmjopen-2022-070479 (PMC10391825; doi:10.1136/bmjopen-2022-070479)
Supplement: Supplementary data [file bmjopen-2022-070479supp001.pdf]

Appendix 1 - Interview Guide District Health Officers

1. In your opinion, what are the main obstacles for the provision of access to healthcare?
2. In your opinion, what are the main obstacles to the provision of surgical care?
3. How do the supply chains for medical equipment work?
4. How do the supply chains for medication work?
5. Which interventions are planned in order to improve access to healthcare, especially to surgical care?
6. Are there campaigns or continuous educational programmes to inform the public about surgical conditions and access to surgical care? Have they proven effective?
7. How much importance is given to the provision of surgery in policy making?
8. What do you see as the most effective measure to increase the surgical workforce?
9. In your opinion, what would be the most effective way for NGOs involved in health care to support the local structures?
10. How could short stays of specialists, e.g. for eye or reconstructive surgery or fistula repair, be integrated into public surgical care for all?

Appendix 2 - Interview Guide Health professionals

Age:

Gender:

Profession:

Place of work:

Position:

Time period of work at current work place:

1. Please tell me about your personal experience with the provision of surgical care.
2. What are the key obstacles that [profession of interviewee] face at this health care facility?
3. What are the main difficulties in the provision of surgical care?
4. How do you deal with these difficulties?
5. How is the aftercare of surgical procedures organised and do you think it works effectively?
6. According to your experience with surgical care, what do you think are the main areas that need to be improved on?
7. What do you perceive as the main difficulties for patients?
8. In your opinion, what is the best way to address these difficulties for the patients?
9. Would you say patients trust in the expertise of health care professionals? How do patients perceive surgical conditions and treatment in your experience?
10. What are areas that you would describe as well-functioning when it comes to surgical care?

Appendix 3 - Interview Guide – Community Members (Former patients)

Age:	When were you operated?
Education:	Surgical condition:
Occupation:	Where were you operated from?
	Length of hospital stay:

1. Please tell me about your experience with surgery.
2. How much time passed from your first symptoms to the diagnosis? And from the diagnosis to the surgical treatment?
3. Were you at any point unsure whether to have surgery and if yes why?
4. Did you consult any party outside of the hospital concerning the health condition you were in?
5. Who took the final decision of whether you would have the surgery?
6. Are you covered by a health insurance?
7. Were there any struggles to reach the hospital?
8. How did the health workers treat you? Were you respected? Were you taken care of?
9. How well did you feel informed about the procedure?
10. How successful was the operation in your opinion?
11. How was the aftercare of the operation? Where was it done? Did you face any struggles taking care of the wound after being discharged from hospital? Did you go through any episodes of extreme pain after the operation?
12. How did your operation change your attitude towards surgery? [This question was amended and changed to: If someone in your family needed to have surgery, what would you advise them?]
13. Throughout the entire process what were the main struggles you faced? In your opinion, which suggestions do you have to improve health facilities in Uganda or your community?
14. In which way did your community support you during that operation and your stay at the hospital?
15. Do you have any questions concerning what we have discussed with you?

Appendix 4 - Interview Guide – Community Members (general population)

Age:

Education:

Occupation:

1. Please tell me about your thoughts on surgery.
2. What do you think are the biggest barriers to surgical care that members of your community face?
3. Do you know where the closest surgical facility is and how long would it take you to get there?
4. What do you think are health problems for which surgery is needed?
5. Do you go to healthcare facilities regularly for check-up or only in case you experience symptoms?
6. How would you estimate the financial impact of a surgical operation on your livelihood?
7. Are you covered by a health insurance?
8. Would you be concerned if you were told by a healthcare provider that you had to have surgery?
9. If yes, what would be your major concerns?
10. Which things would you take into consideration before opting for surgery?
11. In your opinion, are patients treated right by healthworkers at the hospital?
12. How do people in this community support each other in case someone needs to go to the hospital?
13. Do you think consultations with traditional healers are helpful for possible surgical conditions?
14. Do you have traditional healers in your area? If yes, why do you think patients first consult them instead of going to the health facility directly? For the treatment of which cases are these traditional healers best known for?
15. Which measures would improve access to surgical care for your community in your opinion?
