# Supplementary material for: Acceptability of surgical care in Uganda: a qualitative study on users and providers
Source: BMJ Open. 2023 Jul 31;13(7):e070479. doi: 10.1136/bmjopen-2022-070479 (PMC10391825; doi:10.1136/bmjopen-2022-070479)
Supplement: Author's manuscript [file bmjopen-2022-070479.draft_revisions.pdf]

BMJ Open is committed to open peer review. As part of this commitment we make the peer review history of every article we publish publicly available.

When an article is published we post the peer reviewers' comments and the authors' responses online. We also post the versions of the paper that were used during peer review. These are the versions that the peer review comments apply to.

The versions of the paper that follow are the versions that were submitted during the peer review process. They are not the versions of record or the final published versions. They should not be cited or distributed as the published version of this manuscript.

BMJ Open is an open access journal and the full, final, typeset and author-corrected version of record of the manuscript is available on our site with no access controls, subscription charges or pay-per-view fees (http://bmjopen.bmj.com).

If you have any questions on BMJ Open's open peer review process please email info.bmjopen@bmj.com

Acceptability of surgical care in Uganda: A Qualitative Study on Users and Providers

Journal:	*BMJ Open*
Manuscript ID	bmjopen-2022-070479
Article Type:	Original research
Date Submitted by the Author:	08-Dec-2022

Complete List of Authors:	Rauschendorf, Paula; University of Bonn, Section Global Health, Institute of Hygiene and Public Health<br>Nume, Rosette; Makerere University, School of Women and Gender Studies<br>Bruchhausen, Walter; University of Bonn, Section Global Health, Institute of Hygiene and Public Health

Keywords:	SURGERY, Organisation of health services < HEALTH SERVICES ADMINISTRATION & MANAGEMENT, Clinical governance < HEALTH SERVICES ADMINISTRATION & MANAGEMENT, Quality in health care < HEALTH SERVICES ADMINISTRATION & MANAGEMENT

SCHOLARONE™
Manuscripts

I, the Submitting Author has the right to grant and does grant on behalf of all authors of the Work (as defined in the below author licence), an exclusive licence and/or a non-exclusive licence for contributions from authors who are: i) UK Crown employees; ii) where BMJ has agreed a CC-BY licence shall apply, and/or iii) in accordance with the terms applicable for US Federal Government officers or employees acting as part of their official duties; on a worldwide, perpetual, irrevocable, royalty-free basis to BMJ Publishing Group Ltd ("BMJ") its licensees and where the relevant Journal is co-owned by BMJ to the co-owners of the Journal, to publish the Work in this journal and any other BMJ products and to exploit all rights, as set out in our licence.

The Submitting Author accepts and understands that any supply made under these terms is made by BMJ to the Submitting Author unless you are acting as an employee on behalf of your employer or a postgraduate student of an affiliated institution which is paying any applicable article publishing charge ("APC") for Open Access articles. Where the Submitting Author wishes to make the Work available on an Open Access basis (and intends to pay the relevant APC), the terms of reuse of such Open Access shall be governed by a Creative Commons licence – details of these licences and which Creative Commons licence will apply to this Work are set out in our licence referred to above.

Other than as permitted in any relevant BMJ Author's Self Archiving Policies, I confirm this Work has not been accepted for publication elsewhere, is not being considered for publication elsewhere and does not duplicate material already published. I confirm all authors consent to publication of this Work and authorise the granting of this licence.

For peer review only

Acceptability of surgical care in Uganda: A Qualitative Study on Users and Providers

1. Corresponding author
   Paula Kristina Rauschendorf
   Section Global Health
   Institute of Hygiene and Public Health
   University of Bonn
   Bonn, Germany
   p.rauschendorf@outlook.de
   Tel. +1(617)201-1231

2. Rosette Nume
   School of Women and Gender Studies
   Makerere University
   Kampala, Uganda

3. Prof. Dr. med. Walter Bruchhausen Dipl.-Theol. M.Phil.(Glasg.)
   Section Global Health
   Institute of Hygiene and Public Health
   University of Bonn
   Bonn, Germany

Keywords: Health education and promotion, Surgery, Qualitative Study, Health services research

Word count: 5280

For peer review only

Acceptability of surgical care in Uganda: A qualitative study on users and providers

Abstract

Objectives This study was conducted to assess acceptability of surgical care in Eastern Uganda and enable better allocation of resources and to guide health policy towards increased surgical care seeking.

Design This qualitative study utilized semi-structured in-depth interviews that were transcribed and analysed by coding according to grounded theory.

Setting The study was set in Eastern Uganda in the districts of Jinja, Mayuge, Kamuli, Iganga, Luuka, Buikwe and Buvuma.

Participants Interviews were conducted with 32 former surgical patients, 16 community members who had not had surgery, 17 healthcare workers involved in surgical care and 7 District Health Officers or their deputies.

Results The five intersecting categories that emerged were health literacy, perceptions, risks and fears, search for alternatives, care/treatment and trust in healthcare workers. It was also demonstrated that considering the user and provider side at the same time is very useful for a more extensive understanding of surgical care seeking behaviour and the impact of user-provider interactions or lack thereof.

Conclusion While affordability and accessibility are well defined and therefore easier to assess, acceptability is a much less quantifiable concept. This study breaks it down into tangible concepts in the form of five categories, which provide guidance to future interventions regarding surgical care acceptability. We also demonstrated that multiple perspectives are beneficial to understanding the multifactorial nature of health care seeking and provision.

Strengths and limitations

- This study provides multiple perspectives by interviewing both providers and users of surgical care.
- Among the interviewees of each group, diversity was created through purposive sampling.
- This study is limited by its small sample size and geographical area, despite its attempt for representativity.
- Some questions needed re-phrasing after analysis of the first set of interviews following grounded theory due to them being misunderstood

For peer review only

Introduction

In 2015, the Lancet Commission Report on Global surgery highlighted the inequity of the global burden of surgical disease and access to care [1]. Surgery has been found highly effective in reducing the burden of disease and DALYs lost to disability [2]. In connection with Universal Health Coverage (UHC) as a target of the Sustainable Development Goal 3 [3] the question of *accessible*, *acceptable* and *affordable* surgical services is up for discussion.

Uganda has a high unmet need for surgery at a national proportion of more than 10% [4], 28% among the ageing population [5] and unmet paediatric surgical need at 17% [6]. With an average annual population growth rate of 3.0% [7] and increasing life expectancy, the number of patients requiring surgery is expected to rise.

There is a severe mismatch between the necessary and currently available capacity. Health facilities were built for smaller populations with few operating theatres (0.2 major OTs per 100,000 people) [8], leading to overbooked wards [9]. With a surgeon density of 0.73 surgeons per 100,000 persons [6], non-specialised physicians perform most surgical procedures. Shortages of necessities like water and electricity further complicate safe surgical care. Similarly, equipment is often unavailable, broken, or out-dated. Lacking access to blood banks further limits the surgical capacity of Ugandan hospitals [10].

The accessibility of surgical services has been thoroughly researched in Uganda. In 2017, less than 25% of the population could access a facility within 2 hours that performed the Bellwether procedures [11]. Only 62.5% of private hospitals [12] and 56.3% public hospitals [13] were found able to perform all Bellwether procedures. Financial constraints have been a known barrier for patient's access to surgical treatment. Care was found to be unaffordable even at a free government hospital due to expenses of buying commodities [14].

In comparison, acceptability of care has not been a focus of research. Among children with surgical need, for 74.2% formal healthcare was sought. Out of these children, 25% remained untreated because the patient's family did not perceive any surgical need [15]. Research on acceptability of care can provide insights into possible reasons for this and allow for better allocation of resources and more efficient development of health policies towards specific issues.

This study was conducted to explore patients' expectations and dissatisfactions regarding surgical care and to understand underlying causes by considering the perspective of former patients, community members, healthcare professionals and District Health Officers.

Methods

Study design

This qualitative study utilized semi-structured in-depth interviews that were transcribed and analysed by coding according to grounded theory. The interviews were conducted in the districts of Jinja, Mayuge, Kamuli, Iganga, Luuka, Buikwe and Buvuma. Four groups were interviewed: former surgical patients (FP), community members who had not had surgery (CM), healthcare workers involved in surgical care (HW), and the District Health Officers (DHO) of the districts. In each district, the goal was to conduct six interviews with FPs, three interviews with CMs from three different communities, three interviews with HWs, one each from a private for profit (PFP), private non-for-profit (PNFP) and government (public) facility and an interview with the DHO.

Figure 1

This was not possible in all districts due to the lack of facilities in some districts. In this case, either two interviews were conducted at a government facility, or one interview was

For peer review only - http://bmjopen.bmj.com/site/about/guidelines.xhtml

conducted in the district. If possible, different medical cadres were interviewed for each sector to maximise variety and minimize selection bias in accordance with purposive sampling.

Patient and public involvement

There was no patient or public involvement in the study prior to data collection.

Setting

Jinja District and the mentioned surrounding districts are located in South-Eastern Uganda and an estimated population of 2.84 million [16]. Except for Buikwe, which is in Buganda region, the districts lie in Busoga region. The eastern region has a significantly higher poverty rate than the national average. [17]

Data collection

Overall, 72 interviews were conducted from August to September 2019 until theoretical saturation was achieved. Community interviews were conducted in Lusoga. Interviews with HWs and DHOs were conducted in English or Lusoga depending on preference.

The three communities per district were chosen purposively with regards to distance to the next surgery providing health care facility and access to the main road. Community members were then approached and asked whether they had ever had surgery or whether they could point us to a community member who had previously had surgery. This was repeated until two FPs and one CM were recruited.

Interviews with FPs began with an open question about their experience with surgery, followed by more specific questions regarding delays in healthcare seeking, reasons for uncertainty, consultations outside of biomedical healthcare, decision making, the process of their surgical treatment, changes in surgery perception, community support and suggestions for improvement of health facilities.

CMs were asked for their thoughts on surgery and their perceived struggles, health behaviours like regular check-ups, knowledge on surgical conditions, financial impact, health insurance coverage and their concerns if they needed to have surgery.

HWs were asked for their experience in the provision of surgery, including more focused questions around difficulties, their improvement, and patient perceptions.

Interviews with DHOs commenced with a question about obstacles regarding the provision of access to healthcare in general and especially surgical care. Questions regarding supply chains, health education campaigns, the surgical workforce and ways of involvement of NGOs followed.

For full interview guides please refer to Appendix 1-4.

After analysis of the first set of interviews, some questions were rephrased for clarity.

Interviews were audio recorded, transcribed verbatim and anonymized.

Data analysis

Data was analysed using MAXQDA Pro Analytics. Interview transcripts were read thoroughly and coded according to grounded theory by finding the key words in each section of the interviews. Codes were grouped into sub-categories and sorted into larger categories.

Results

Interviews were conducted with 32 FP, 16 CM, 17 HW and 7 DHO or their deputies.

For peer review only

During the analysis, five dimensions of acceptability of surgical care emerged. *Health literacy* impacted the healthcare seeking behaviour as well as the frame of expectations regarding surgical care, which was also shaped by *fears and perceptions of surgery*. Surgical care was also compared to other means of healthcare in a search for *alternatives*. Trust in the *healthcare worker* as well as *care and treatment* emerged as categories that played a major part in acceptability.

Health literacy: impact and improvement

Low levels of health literacy impacted healthcare seeking negatively according to many participants from all groups. A DHO lamented that patients did not know the “timely need for coming for healthcare”. HWs supported this and explained that “people don’t know that they require surgery” (surgeon, PNFP) and that “they don’t even know the impact of their condition” (MD, PNFP). An FP stated that during the operation of an intestinal obstruction one year before, a mass was found in his spleen. Since then, he had not returned to the health facility to get the mass checked.

Many FPs reported waiting for a long time before seeking care, as one said: “I over delayed. As you know when you over delay, that hernia gradually grows. I delayed a bit. I think it was around [...] 10 years” (FP, PFP). Along with other participants, another FP attributed the delay to his “ignorance, because I wasn’t aware the illness needed a medical treatment” (FP). Some participants described self-medicating, as in this example: “When I am sick, I just buy medicine of 2000 to 3000 shillings and if that sickness doesn’t respond to the treatment, I continue changing the type of treatment until I get better.” (CM)

Low levels of general education were discussed, as “perceptions are about education” (MO, public). A HW described struggling to find a comprehensible way to explain surgical conditions to the patients. CMs similarly said: “after taking blood samples from us, there’s nothing you can understand” (CM) and “those who never went to school don’t know anything and you have to believe all what the healthcare workers tell you” (CM).

While many CMs demonstrated knowledge on obstetric conditions and hernia, other conditions such as goitre, ulcer, and cancer were less frequently mentioned. A HW attributed this to the fact that “surgery doesn’t have enough advocacy, but obstetric, emergency obstetric care has received a lot of advocacy and a lot of funding” (MO, public). He described the increase in health education campaigns around obstetric emergencies had led to women “coming to the facilities, seeking care, surgical care compared to general surgery.” (MO, public). Another HW confirmed the importance of such campaigns for recruiting patients, “you will not find a health education anywhere talking about cleft palate and cleft lip until there is a camp somewhere and they are looking for those patients.” (MO, PNFP)

Therefore, more health education specific to surgery was proposed to improve surgical care seeking. Suggestions included campaigns broadcasted via radio and televisions as well as outreaches and village health teams (VHTs) “to identify some of these complications – the illnesses – and educate, give some health education and refer these people to where they can get services” (Surgeon, PFP). One HW raised concerns that educating the population had to be linked with building surgical capacity to accommodate the anticipated increase in surgical patients.

Perceptions and Fears of Surgery

Surgery was perceived by CMs and FPs as a way of “reducing the sickness that is bothering you” (FP, PNFP) by “cutting you open to see the cause of sickness and remove the unwanted stuff” (FP, PFP), oftentimes with urgency: “the patient is in a critical and not understandable condition, then why can that patient not be taken to be operated” (CM). Surgical care was described as a last resort by CMs and FPs, often only an option “when the

For peer review only

condition that calls for the operation is very extremely severe, when the pain is so so so beyond, without giving you time to eat, drink or walk, I mean when the illness is at its maximum, then there you can decide to go for the operation" (CM).

The risk associated with surgery was often named as "you can come back or not come back" (CM). A surgeon from a PNFP facility attributed these sceptical attitudes to the fact that "in the past the results from surgery have not been good. It has been between life and death." One of the DHOs mentioned that cases with complications are overly emphasised causing a prevalent misconception that surgery is unsafe. Seeming medical malpractice in anaesthesia was described by one FP: "When I was still half sedated, they cut me but the way they pulled my scrotum with much force I felt terrible pain in the intestines" (FP, PFP).

Participants mentioned the fear that surgeons would leave instruments or swabs in the patient with possible complications and re-operations. One recounted that the people "normally say that when they go for surgery, they make you more disabled not allowed to do certain work" (CM). Another example was the fear that putting a patient "on oxygen means he's going to die" (nurse, PNFP). Also, there were "rumours that healthcare workers steal certain organs from the patients" (CM) which led to fear among the community. One FP suggested having a relative of the patient in the theatre to "see the procedures of the operation and what they have removed from the patient" (FP, public).

One midwife (PFP) saw an increase in trust after successful operations, which was also apparent in some interviews with FPs: "healthcare providers can operate and after the operation you can wake up and resume with normal walking, healthy person, and the illness is taken away you can become better, and you live a healthy life." (FP, PNFP) Another FP described how she was asked after her surgery at a PFP facility "not to scare my fellow women that the health facility was a terrible place; they told me to advise my friends to go for medical procedures". This demonstrates that staff feels the need for improving the reputation of surgical treatment. As a way of counteracting patient fear, effective pre-operative counselling was suggested by FPs and healthcare providers.

Understanding of Illness and the Search for Alternatives

Surgical conditions as seen from a biomedical perspective were mostly regarded as necessitating biomedical treatment and many participants named biomedical facilities as their only source of healthcare. Patients mostly became aware that their condition was surgical after it had been identified as such by a healthcare worker. One FP stated: "sickness that is meant for operation, still goes for operation, there is no alternative" (FP, PNFP). In contrast to the view that surgical conditions always necessitate surgery, one medical officer described that pertaining to surgical diagnosis "they [the patients] think there is something that you have not done. They think you should have given some medication for that condition."

Herbal medicine, for example was described as a way of preventing surgery under certain circumstances: "I was to have a normal delivery if I used the local herbs" (FP, public). It was only mentioned in the context of chronic conditions like hernia, not as an alternative in emergency or trauma cases. An FP described "someone went to those healers and was given herbs and his hernia disappeared, so I also went there and used those herbs for one day but when I noticed no improvement, I gave up on them." (FP, PFP). Another example was given by an FP with haemorrhoids, who was later operated: "when I applied the herbs, the piles went back but after some time, it re-occurred, and when I asked them about that, they told me that, the piles can only be healed with traditional medicine and so I continued with that medicine." (FP, PFP) HWs expressed frustration but blamed it on the "traditional

For peer review only

healers or herbalists who keep misguiding or giving false information to our clients" (PFP, surgeon) and the lack of knowledge concerning surgical conditions.

Similarly, when asked about traditional healers in the area, many respondents voiced distrust because they would not "reveal that your condition needs surgical treatment. When they want money, they just divert you from seeking medical care, yet they can't treat the problem at hand, they just confuse patients by assuring them how they can manage their health condition in just minutes." (CM) However, some participants saw fault with the patients that sometimes "cannot differentiate a medical problem from a traditional one" (CM) and "think that they have been bewitched, even if the sickness is not traditional" (FP). This shows an understanding of ill-health and healing where two spheres are separated for different illnesses and their corresponding healthcare, i.e. an alternative between "medical" and "traditional".

Differing from this understanding of two distinct types of illness and treatment, a CM argued "those other things of bewitching can come later after going to the health facility" (CM). Here the common view of complementary functions is expressed that biomedicine may – even quickly – remove the damage like hernia, but not the underlying cause like bewitchment, as one said: "Can a healthcare worker tell you issues of the home? […] But it is the traditional healer to tell you the facts. The healthcare worker can suspect the cause of illness and tell the patient that maybe the patient delayed getting treatment, but when you go to these ones, they can tell you." (CM).

Care and Treatment

The received care was commonly described as good, defined differently by FPs e.g. not being mistreated or being spoken to "in a friendly manner" (FP, PNFP). Others voiced more specific expectations: "to be cared for, is when the healthcare worker comes in the ward and greets you asking about how the night was, how you feel, the condition of the baby and other issues like that." (FP, public) For others the focus was more on the medical aspects like receiving "timely treatment" (FP, PFP).

The type of care received was commonly attributed to the patient's financial situation, where paying the asked amount was linked to good care and medical treatment, whereas patients with financial constraints were often mentioned to receive worse treatment. Other FPs attributed the good care they received to personal relationships with the healthcare workers. One FP reported that "they respected me because first of all I am educated and I interacted with them in English during all the chats" (FP, PNFP).

Despite general satisfaction, some complaints were voiced like going through episodes of extreme pain after surgery, some to the extent, that it would prevent future surgical care seeking. Other FPs mentioned rude treatment by healthcare workers, for example, a female FP was on a trolley to the OR for a C-section when she "felt something like a baby coming out then I widened my legs as if I was going to deliver, then the nurse slapped me for widening the legs, and exposing my nakedness to the people" (FP, public). The nurse was later retributed for her actions. A respondent who had not had surgery lamented: "if you are lucky enough and your patient is operated, they carelessly stich and just dump him/her there for you. They are always not concerned with the aftereffects of the operation, it's you to care for the patient." (CM)

This alludes to the important role of informal caregivers in Ugandan healthcare. "They carry the burden, the financial, emotional, and so on" (surgeon, public). Their importance was highlighted multiple times throughout interviews, for example when one FP said, "if you have

For peer review only

no effective caregiver, it's a big challenge." (FP, public). The informal caregiver has different responsibilities, mainly taking care of the basic needs of patients, while at the hospital and to provide emotional support. One FP stated, that when patients did not have their treatment readily prepared by their informal caregiver nurses "just pass to another patient" (FP, public).

The "lack of proper trained surgical nursing care" (medical superintendent) led to some surgeons feeling "let down by the postoperative care" (MO, public). A nurse explained: "we are like 2 or 3 on duty and patients are many and we have to attend to them, sometimes you find that you have not reached up to that peak of caring for the patient" (nurse, PFNP). Therefore, sometimes "responsibilities channel to the relatives" (MO, public) that should be carried out by medical personnel. A surgeon noted that this led to problems in the aftercare of operations. HWs also noted that the lack of separate surgical wards was a challenge to providing specialised care. To counteract these challenges, more training, a separate surgical ward and an increase in staff were suggested.

Follow-up of operations was another theme that emerged. Some FPs said they had not been asked back for follow-up. HWs commonly said that review dates were scheduled with patients, but attendance varied. This was a challenge for surgical staff, as doctors were then "not able to monitor them for any complications" (Surgeon, PFP). Varying commitment from HWs was apparent, with some recording patients' numbers and calling to check up and some visiting their patients at home. A surgeon suggested that regular "field visits" (Surgeon, PFP) could improve follow-up.

Healthcare workers: Trust and Misbehaviour

Closely linked to care and treatment was patient-healthcare worker relationship. As one CM stated: "the healthcare worker is the overall in this thing, he/she is the key to everything". Trust and lack thereof were commonly mentioned.

Most interviewed HWs agreed that they felt trusted by patients. Some FPs described a trustful relationship, with one mentioning being taken care of by a doctor off duty and another calling the doctor a "saviour" (FP, public). One medical officer (public) differentiated: "Those who come, trust us. What they don't trust is the system. What they don't trust is their government". Contrastingly, distrust in the surgical skills of healthcare workers was also mentioned with one FP saying: "most of the healthcare workers are young boys, we don't trust them" (FP). This is caused by the regulation that demands service in remote communities in the first years after graduation. One doctor (PFP) highlighted that this was a major difference to traditional healers, as they "tend to give more time to their patients actually and convince them adequately". The overwhelming workload and little time spent with the patient were given as reasons for the lack of trust in the healthcare system and HWs.

HWs were expected to "first think about people's health, then think about money" (CM) because "there are rewards in heaven" (CM). Work ethics of HWs were a concern to participants from the community, who described nurses and doctors commonly asked for informal payments. One FP described being asked "to pay her 60,000 shillings for quick treatment and we go home instead of being admitted in the ward" (FP, public). Some mentioned, HWs would let the patient die if they could not pay: "There was that first one I saw, he had even refused to operate me, and if the second one never showed up, I was going to die" (FP, public). Participants suggested to raise salaries of HWs, so they would stop demanding informal payments.

Medical personnel generally described their own work ethics as high, with one medical officer calling it a "commitment to serve". One doctor described the line between ethics and business at a PNFP "sometimes it is necessary, where you see that life is more important".

For peer review only

Nevertheless, many saw issues with motivation and work morale in the health workforce in general, attributing it to frustration due to poor salaries, long working hours, poorly equipped facilities, and little opportunity for continued education and mentorship. To improve morale through mentorship, one DHO suggested. Many saw scholarships for further education as important. Training programmes through foreign exchange or as part of surgical camps were seen as beneficial for both sides: “Probably you might come with the skills where you come from, but the context differs. Then you also have something to learn with us, from us working with limited resources.” (DHO)

Discussion

In providing surgical care to communities, like in Uganda, factors beyond accessibility and affordability necessitate careful consideration. While some commonly used models like the Behavioural Model of Health Service Use by Andersen [18] consider mostly the user side, this study aims to showcase both the user and provider side and their interaction regarding surgical cases. This provides a better understanding of reasons for the fits and misfits [19] between user expectations and provided surgical services.

The five areas that impacted acceptability regarding surgical care found in this study were: health literacy, perceptions of surgery, the search for alternatives, care and treatment and the relationship to the healthcare worker. These categories, however, were not clearly separable and often overlapped.

Higher health literacy has been shown to increase acceptance of surgical care for urinary incontinence [20]. Participants of our study, especially on the provider side also viewed health literacy on surgical conditions impactful for improving surgical care seeking. It should be a focus of future interventions, as already suggested by Butler et al. in a study which yielded lack of perceived need as reason for 25% of children with surgical needs to remain untreated [15]. General education and literacy were also discussed by participants as enabling patients to make better decisions on healthcare seeking for surgical conditions, which was also found by Fuller et al. and Davé et. al [21].

A thorough understanding of the perceptions of surgery and concept of illness is necessary to provide acceptable care. The question of what is at stake for those people most concerned by a surgical condition needs to be addressed for meeting important expectations and creating trust. Understandably sceptical attitudes due to former negative experiences with formal education and biomedical healthcare [22] as well as unfavourable surgical outcomes [23] have a large impact on surgical healthcare seeking.

While the surgical treatment was often described as successful by patients, negative perceptions were commonly mentioned. Risks and fears related to surgical interventions act as a barrier for their uptake. Perceptions were also shaped by rumours, like concerns of organ stealing. Rumours like this have existed in many African societies and have been discussed extensively by White [24], Scheper-Hughes [25] and Musambachime [26]. Other rumours noted were specific to surgical care and to proceedings of an operating room. Improving health literacy, setting a focus on communication with patients and improved education prior to surgery could play an important part in counteracting rumours. It could also reduce self-medication and change the role of surgery as a last resort or for emergencies only.

With 4.0% of rural children with surgical conditions not receiving care due to fear [27], trust and positive interactions with surgical care providers are important. While most healthcare personnel felt trusted by patients and many patients voiced trust, there was some scepticism towards surgical skills particularly of medical officers. Similar concerns about seniority and skill level were voiced by participants in study by Mwaka et. al suggesting that only senior

and skilled doctors are fit to successfully operate cancer patients [28]. Due to lack of specialists, non-specialised personnel commonly conducts surgery [29] in Uganda.

Participants in our study, especially medical officers, wished for more mentorship and further specialisation. This would also provide an opportunity for more training on interpersonal skills to improve communication with patients, as suggested by Bohren et. al [30]. Surgical camps were suggested as possible training opportunities. Modern technology was also proposed to improve mentorship, where medical officers in remote areas would be able to consult with colleagues in Uganda or abroad via telecommunication.

Most FPs and CMs recognized the need for operative care in case of surgical conditions. These findings align with results from a previous study investigating surgery beliefs of lay people, where surgery was seen as “the appropriate healing method”, although there was also “fear of negative outcome of surgery” [28]. In our study, some participants mentioned attempting alternative treatments under the category of “traditional medicine”, which was also a common theme among providers. Participants from communities often agreed there were indications for traditional medicine, however rarely including surgical conditions like hernia.

Many participants from all groups highlighted the dangers of misdiagnosis and delay of treatment, which has been discussed by WHO in their traditional medicine strategy [31]. Proof of inadequate treatment by traditional healers was found among the ageing population in Uganda with surgical conditions [5], as well as an increased likelihood of untreated surgical conditions in paediatric patients who had consultations with traditional healers [15]. Yet healthcare providers observed that traditional healers often spend more time with patients which could be due to the ratios of 1 traditional healer per population of 500, compared to 1 medical doctor per 25000 people in Uganda [32]. Participants also discussed their role as holistic healers, which has formerly been attributed to their ability to re-establish “social and emotional equilibrium” [33] and the provision of “assurance, definition in the midst of despair, hopeful action” [34]. Some of these features could possibly be a starting point for developing a healthcare system encompassing the underlying needs of patients answered by traditional medicine.

Overall, patients in this study were satisfied with the services they received. However, many attributed the type of care they received to their financial situation, their level of education or their relationship with the healthcare worker. Similarly, in a study on patients’ ability to exercise their rights during their stay at a public hospital, many felt the care they received depended on “their ability to bribe the HW with a tip, their socio-economic class, political status and affiliation, prior knowledge and relationship to the HW, and tribal favouritism by HWs.”[35] Stephens et al. found that the economic situation prior to injury was not associated with access to orthopaedic trauma surgery in Uganda, but social capital and financial leveraging increased the likelihood of receiving surgical care.[36] Participants in our study described similar situations. They linked these behaviours to work ethics of HWs. While HWs felt their own motivation was high, many described generally low morale among the health workforce. Reasons given were low salaries, difficult work environments and lack of mentorship.

Participants also described negative interactions between healthcare providers and patients. Being treated harshly was a concern for patients, as well as feeling neglected, similar to former findings in Benin [23]. The power differential between healthcare providers and patients is sometimes used against patients [37]. This is in part due to lack of negotiating power because of “illiteracy, language barriers, low socio-economic status, feeling inferior to HWs and perceived favour to access free services”[35].

For peer review only

Some FPs described situations which could be interpreted as malpractice or errors in surgical care. A study on general medical errors found how they occurred and that healthcare professionals were mostly aware of them [38]. In regards to surgical errors, “inappropriate pre-operative management, omitting preoperative investigations and omitting post-operative notes“ [39] were most commonly found by reviewing patient records. A lack of error reporting on medication errors was also found by Kiguba et. al. [40].

Post-surgical care and follow-up were seen as major challenges by patients and healthcare providers. Lack of trained nursing staff lead to task shifting to informal caregivers, as highlighted by participants. In post-operative caregiving, informal caregivers often lack the necessary training, education and understanding of medical conditions necessary to provide the best care possible. Formerly proposed caregiver training to increase capacity of post-surgical care [41] was not suggested by participants in this study, who laid more focus on increasing the surgical workforce.

Previous research suggests that receiving follow-up care is strongly dependent on socio-economic status and distance to access points [41]. Our findings support this. Healthcare professionals in our study suggested taking phone numbers of patients and making fixed appointments to combat this problem. Home visits were mentioned as ideal, but not feasible. A study on maternal and new-born care practices also suggests that a combination of home visits by VHTs and mobile phone consultations improve care.[42] Interventions linked to VHTs should be strengthened and possibly expanded to include regular basic health check-ups to facilitate earlier interventions on complications and remissions.

Limitations

In its aim to provide a broad overview of perspectives on surgical care, this study is limited by its small sample size and geographical area, despite its attempt for representativity. Concerning both, reliability and validity, some questions needed re-phrasing after analysis of the first set of interviews following grounded theory due to them being misunderstood.

Conclusion

For promoting the use of surgical care, this study highlighted the importance of studying the views of users, providers and local policymakers in order to provide more all-encompassing insights into expectations, provided services and the causes of possible misfits and delays. It also breaks down acceptability of surgical care into more tangible targets to guide future policies. Expectations of surgical care emerged to be shaped by health literacy and perceptions of illness and surgery. Extensive information and health education campaigns on surgical conditions could improve healthcare seeking and empowerment of patients and their relatives. A closer look at reasons for alternative treatments sought out by patients provides a foundation for future interventions. The care and treatment at large as well as the relationship with the healthcare worker impacted acceptability. In this area negative experiences by patients could be avoided by increased training of medical personnel, improving on interpersonal skills, and encouraging a respectful environment in hospitals and health. The needs of providers should be taken into consideration to improve motivation and workplace satisfaction. Structural improvements and an increase in qualified staff are the basis for a work environment in which acceptability can be given the necessary attention. Finally, our findings suggest the need for more extensive research on the underlying mechanisms of acceptability of surgical care, as an important foundation to tackle delays in surgical care seeking.

For peer review only

Ethical considerations

Permission to conduct the interviews was obtained from the Ugandan National Council for Science and Technology after receiving ethical clearance from Mildmay Uganda Research Ethics Committee under #REC REF 0102-2019. For interviews with DHOs permission was given by the Ministry of Health. Furthermore, we asked permission of DHOs to conduct interviews with HW in their district. All participants provided informed consent to be eligible to participate in this study.

Data Sharing statement

We provided all relevant excerpts within the manuscript as raw data cannot be shared publicly due to the significant and valid confidentiality concerns of interviewees, small sample size, and ethics approval requirements.

Conflict of Interest

The author(s) certify that they have no conflict of interest in the subject matter or materials discussed in this manuscript.

Funding

The study was funded by the Department of Global Health at the Bonn University Hospital.

Author Contributions

PR, a German medical student at the time of the study, and WB, a professor for Global Health at the University of Bonn, developed the study design and the interview guides. Data collection was carried out collectively by PR and RN, a master's student at the School of Women and Gender Studies. PR then conducted the data analysis and prepared the manuscript. WB oversaw all aspects of this research. All authors contributed to the article and approved the submitted version.

Acknowledgements

We thank Global Health at the Bonn University Hospital for the financial support of this study, Ando-Modular aid e.V. for the inspiration for this study, Hatiika Namyalo for helping us navigate the Ugandan health care system and Grace Meara for helpful discussions.

For peer review only

References

1 Meara JG, Leather AJM, Hagander L, et al. Global Surgery 2030: evidence and solutions for achieving health, welfare, and economic development. *Lancet* 2015;386(9993):569–624. http://www.sciencedirect.com/science/article/pii/S014067361560160X.

2 WHO Executive Board. Strengthening emergency and essential surgical care and anaesthesia as a component of universal health coverage: Report by the Secretariat 2014.

3 UN General Assembly. Transforming our World: The 2030 Agenda for Sustainable Development 2015.

4 Farber SH, Vissoci JR, Tran TM, et al. Geospatial Analysis of Unmet Surgical Need in Uganda: An Analysis of SOSAS Survey Data. *World journal of surgery* 2017;41(2):353–63.

5 Tran TM, Fuller AT, Butler EK, et al. Surgical need among the ageing population of Uganda. *Afr Health Sci* 2019;19(1):1778–88.

6 Butler EK, Tran TM, Nagarajan N, et al. Epidemiology of pediatric surgical needs in low-income countries. *PloS one* 2017;12(3):1-9. doi:10.1371/journal.pone.0170968 [published Online First: 4 March 2017].

7 Uganda Bureau of Statistics. 2020 Statistical Abstract.

8 Linden AF, Sekidde FS, Galukande M, et al. Challenges of surgery in developing countries: a survey of surgical and anesthesia capacity in Uganda's public hospitals. *World journal of surgery* 2012;36(5):1056–65.

9 Nwanna-Nzewunwa OC, Ajiko MM, Kirya F, et al. Barriers and facilitators of surgical care in rural Uganda: a mixed methods study. *The Journal of surgical research* 2016;204(1):242–50. doi:10.1016/j.jss.2016.04.051 [published Online First: 28 July 2016].

10 Albutt K, Yorlets RR, Punchak M, et al. You pray to your God: A qualitative analysis of challenges in the provision of safe, timely, and affordable surgical care in Uganda. *PloS one* 2018;13(4):1-16. doi:10.1371/journal.pone.0195986 [published Online First: 18 April 2018].

11 Albutt K, Namanya D, Anderson G, Kayima P. Uganda. In: Johnson W, Lin Y, Mukhopadhyay S, Meara J, ed. Surgical care systems strengthening: developing national surgical, obstetric and anaesthesia plans. Geneva: World Health Organization 2017:4–7.

12 Albutt K, Drevin G, Yorlets RR, et al. 'We are all serving the same Ugandans': A nationwide mixed-methods evaluation of private sector surgical capacity in Uganda. *PLoS One* 2019;14(10):e0224215.

13 Albutt K, Punchak M, Kayima P, et al. Access to Safe, Timely, and Affordable Surgical Care in Uganda: A Stratified Randomized Evaluation of Nationwide Public Sector Surgical Capacity and Core Surgical Indicators. *World J Surg* 2018;42(8):2303–13.

14 Anderson GA, Ilcisin L, Kayima P, et al. Out-of-pocket payment for surgery in Uganda: The rate of impoverishing and catastrophic expenditure at a government hospital. *PloS one* 2017;12(10):1-13.

15 Butler EK, Tran TM, Fuller AT, et al. Quantifying the pediatric surgical need in Uganda: results of a nationwide cross-sectional, household survey. *Pediatric surgery international* 2016;32(11):1075–85. doi:10.1007/s00383-016-3957-3 [published Online First: 12 September 2016].

16 Uganda Bureau of Statistics. Projected population by district (2015-2030) 2019.

17 Uganda Bureau of Statistics, UNICEF, World Bank. Poverty Maps of Uganda: Mapping the Spatial Distribution of Poor Households and Child Poverty Based on Data from the

For peer review only

2016/17 Uganda National Household Survey and the 2014 National Housing and Population Census. Technical Report 2019.
18 Andersen R, Davidson P. Improving access to care in America: Individual and contextual indicators. *Changing the US Health Care System: Key Issues in Health Services Policy and Management* 2014;2007.
19 Penchansky R, Thomas JW. The concept of access: definition and relationship to consumer satisfaction. *Medical care* 1981;19(2):127–40.
20 Biyik I, Usturali Mut AN, Albayrak M, et al. Effect of health literacy on help-seeking behavior: A comparison of patients accepting surgery and refusing surgery for urinary incontinence. *J Gynecol Obstet Hum Reprod* 2021;50(4):101908.
21 Davé DR, Nagarjan N, Canner JK, et al. Global Burden of Craniofacial Disorders: Where Should Volunteering Plastic Surgeons and Governments Focus Their Care? *J Craniofac Surg* 2020;31(1):121–24.
22 Raykar NP, Yorlets RR, Liu C, et al. The How Project: understanding contextual challenges to global surgical care provision in low-resource settings. *BMJ Global Health* 2016;1(4):e000075. doi:10.1136/bmjgh-2016-000075 [published Online First: 16 December 2016].
23 Close KL, Christie-de Jong FTE. Lasting impact: a qualitative study of perspectives on surgery by adult recipients of free mission-based surgical care in Benin. *BMJ Open* 2019;9(11):e028235. doi:10.1136/bmjopen-2018-028235 [published Online First: 7 November 2019].
24 White L. Speaking With Vampires: Rumor and History in Colonial Africa. Berkeley, Calif.: University of California Press 2000.
25 Scheper-Hughes N. Parts unknown. *Ethnography* 2004;5(1):29–73.
26 Musambachime MC. The Impact of Rumor: The Case of the Banyama (Vampire Men) Scare in Northern Rhodesia, 1930-1964. *The International Journal of African Historical Studies* 1988;21(2):201.
27 Bearden A, Fuller AT, Butler EK, et al. Rural and urban differences in treatment status among children with surgical conditions in Uganda. *PLoS One* 2018;13(11):e0205132.
28 Mwaka AD, Okello ES, Wabinga H. Perceptions and beliefs of lay people from northern Uganda regarding surgery for diagnosis and treatment of cervical cancer. *Psychooncology* 2018;27(8):1965–70.
29 Baine SO, Kasangaki A, Baine EMM. Task shifting in health service delivery from a decision and policy makers' perspective: a case of Uganda. *Hum Resour Health* 2018;16(1):1–20. doi:10.1186/s12960-018-0282-z [published Online First: 2 May 2018].
30 Bohren MA, Titiloye MA, Kyaddondo D, et al. Defining quality of care during childbirth from the perspectives of Nigerian and Ugandan women: A qualitative study. *Int J Gynaecol Obstet* 2017;139 Suppl 1:4–16. doi:10.1002/ijgo.12378 [published Online First: 7 December 2017].
31 World Health Organization. WHO traditional medicine strategy 2014 - 2023. Genève: WHO 2013.
32 Abdullahi AA. Trends and challenges of traditional medicine in Africa. *Afr J Tradit Complement Altern Med* 2011;8(5 Suppl):115–23. doi:10.4314/ajtcam.v8i5S.5 [published Online First: 3 July 2011].
33 Hillenbrand E. Improving Traditional-Conventional Medicine Collaboration: Perspectives from Cameroonian Traditional Practitioners. *Nordic Journal of African Studies* 2006;15.
34 Janzen JM, Arkinstall W. The quest for therapy: Medical pluralism in Lower Zaire. Berkeley, Calif.: Univ. of California Press 1978.
35 Kagoya HR, Kibuule D, Mitonga-Kabwebwe H, et al. Awareness of, responsiveness to and practice of patients' rights at Uganda's national referral hospital. *Afr J Prim Health Care Fam Med* 2013;5(1).

For peer review only

36 Stephens T, Mezei A, O'Hara NN, et al. When Surgical Resources are Severely Constrained, Who Receives Care? Determinants of Access to Orthopaedic Trauma Surgery in Uganda. *World J Surg* 2017;41(6):1415–19. https://pubmed.ncbi.nlm.nih.gov/28097413/.
37 Fonn S, Mtonga AS, Nkoloma HC, et al. Health providers' opinions on provider-client relations: results of a multi-country study to test Health Workers for Change. *Health Policy Plan* 2001;16 Suppl 1:19–23.
38 Mauti G, Githae M. Medical error reporting among physicians and nurses in Uganda. *Afr Health Sci* 2019;19(4):3107–17. https://pubmed.ncbi.nlm.nih.gov/32127887/.
39 Katongole Simon Peter, Robert Anguyo, Ddm Onzima, et al. Common Medical Errors and Error Reporting Systems in Selected Hospitals of Central Uganda 2015.
40 Kiguba R, Waako P, Ndagije HB, et al. Medication Error Disclosure and Attitudes to Reporting by Healthcare Professionals in a Sub-Saharan African Setting: A Survey in Uganda. *Drugs Real World Outcomes* 2015;2(3):273–87.
41 Barton SJ, Sandhu S, Doan I, et al. Perceived barriers and supports to accessing community-based services for Uganda's pediatric post-surgical population. *Disabil Rehabil* 2019:1–12.
42 Mangwi Ayiasi R, Kolsteren P, Batwala V, et al. Effect of Village Health Team Home Visits and Mobile Phone Consultations on Maternal and Newborn Care Practices in Masindi and Kiryandongo, Uganda: A Community-Intervention Trial. *PLoS One* 2016;11(4):e0153051. doi:10.1371/journal.pone.0153051 [published Online First: 21 April 2016].

Figure Legend

Figure 1. Sampling strategy per district. The goal was to conduct 6 interviews with FPs, 3 interviews with CMs from 3 different communities, 3 interviews with HWs (one each from a PFP, PNFP and public facility) and 1 interview with the DHO.

For peer review only

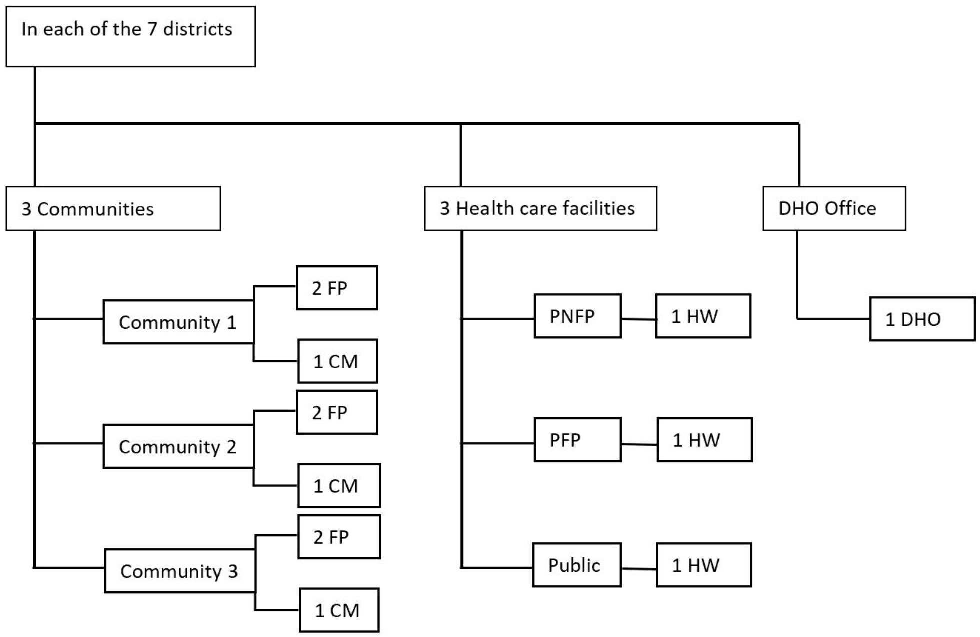

Sampling strategy per district. The goal was to conduct 6 interviews with FPs, 3 interviews with CMs from 3 different communities, 3 interviews with HWs (one each from a PFP, PNFP and public facility) and 1 interview with the DHO.

114x75mm (300 x 300 DPI)

Appendix 1 - Interview Guide District Health Officers

1. In your opinion, what are the main obstacles for the provision of access to healthcare?
2. In your opinion, what are the main obstacles to the provision of surgical care?
3. How do the supply chains for medical equipment work?
4. How do the supply chains for medication work?
5. Which interventions are planned in order to improve access to healthcare, especially to surgical care?
6. Are there campaigns or continuous educational programmes to inform the public about surgical conditions and access to surgical care? Have they proven effective?
7. How much importance is given to the provision of surgery in policy making?
8. What do you see as the most effective measure to increase the surgical workforce?
9. In your opinion, what would be the most effective way for NGOs involved in health care to support the local structures?
10. How could short stays of specialists, e.g. for eye or reconstructive surgery or fistula repair, be integrated into public surgical care for all?

For peer review only

For peer review only - http://bmjopen.bmj.com/site/about/guidelines.xhtml

Appendix 2 - Interview Guide Health professionals

Age:

Gender:

Profession:

Place of work:

Position:

Time period of work at current work place:

1. Please tell me about your personal experience with the provision of surgical care.
2. What are the key obstacles that [profession of interviewee] face at this health care facility?
3. What are the main difficulties in the provision of surgical care?
4. How do you deal with these difficulties?
5. How is the aftercare of surgical procedures organised and do you think it works effectively?
6. According to your experience with surgical care, what do you think are the main areas that need to be improved on?
7. What do you perceive as the main difficulties for patients?
8. In your opinion, what is the best way to address these difficulties for the patients?
9. Would you say patients trust in the expertise of health care professionals? How do patients perceive surgical conditions and treatment in your experience?
10. What are areas that you would describe as well-functioning when it comes to surgical care?

For peer review only

Appendix 3 - Interview Guide – Community Members (Former patients)

Age:

Education:

Occupation:

When were you operated?

Surgical condition:

Where were you operated from?

Length of hospital stay:

1. Please tell me about your experience with surgery.
2. How much time passed from your first symptoms to the diagnosis? And from the diagnosis to the surgical treatment?
3. Were you at any point unsure whether to have surgery and if yes why?
4. Did you consult any party outside of the hospital concerning the health condition you were in?
5. Who took the final decision of whether you would have the surgery?
6. Are you covered by a health insurance?
7. Were there any struggles to reach the hospital?
8. How did the health workers treat you? Were you respected? Were you taken care of?
9. How well did you feel informed about the procedure?
10. How successful was the operation in your opinion?
11. How was the aftercare of the operation? Where was it done? Did you face any struggles taking care of the wound after being discharged from hospital? Did you go through any episodes of extreme pain after the operation?
12. How did your operation change your attitude towards surgery? [This question was amended and changed to: If someone in your family needed to have surgery, what would you advise them?]
13. Throughout the entire process what were the main struggles you faced? In your opinion, which suggestions do you have to improve health facilities in Uganda or your community?
14. In which way did your community support you during that operation and your stay at the hospital?
15. Do you have any questions concerning what we have discussed with you?

For peer review only - http://bmjopen.bmj.com/site/about/guidelines.xhtml

Appendix 4 - Interview Guide – Community Members (general population)

Age:

Education:

Occupation:

1. Please tell me about your thoughts on surgery.
2. What do you think are the biggest barriers to surgical care that members of your community face?
3. Do you know where the closest surgical facility is and how long would it take you to get there?
4. What do you think are health problems for which surgery is needed?
5. Do you go to healthcare facilities regularly for check-up or only in case you experience symptoms?
6. How would you estimate the financial impact of a surgical operation on your livelihood?
7. Are you covered by a health insurance?
8. Would you be concerned if you were told by a healthcare provider that you had to have surgery?
9. If yes, what would be your major concerns?
10. Which things would you take into consideration before opting for surgery?
11. In your opinion, are patients treated right by healthworkers at the hospital?
12. How do people in this community support each other in case someone needs to go to the hospital?
13. Do you think consultations with traditional healers are helpful for possible surgical conditions?
14. Do you have traditional healers in your area? If yes, why do you think patients first consult them instead of going to the health facility directly? For the treatment of which cases are these traditional healers best known for?
15. Which measures would improve access to surgical care for your community in your opinion?

For peer review only

Standards for Reporting Qualitative Research (SRQR)*

http://www.equator-network.org/reporting-guidelines/srqr/

Title and abstract

	Page/line no(s).
**Title** - Concise description of the nature and topic of the study Identifying the study as qualitative or indicating the approach (e.g., ethnography, grounded theory) or data collection methods (e.g., interview, focus group) is recommended	3/3-4
**Abstract** - Summary of key elements of the study using the abstract format of the intended publication; typically includes background, purpose, methods, results, and conclusions	3/9-32

Introduction

**Problem formulation** - Description and significance of the problem/phenomenon studied; review of relevant theory and empirical work; problem statement	4/5-38
**Purpose or research question** - Purpose of the study and specific objectives or questions	4/40-42

Methods

**Qualitative approach and research paradigm** - Qualitative approach (e.g., ethnography, grounded theory, case study, phenomenology, narrative research) and guiding theory if appropriate; identifying the research paradigm (e.g., postpositivist, constructivist/ interpretivist) is also recommended; rationale**	4/47-48
**Researcher characteristics and reflexivity** - Researchers' characteristics that may influence the research, including personal attributes, qualifications/experience, relationship with participants, assumptions, and/or presuppositions; potential or actual interaction between researchers' characteristics and the research questions, approach, methods, results, and/or transferability	13/28-33
**Context** - Setting/site and salient contextual factors; rationale**	5/12-16
**Sampling strategy** - How and why research participants, documents, or events were selected; criteria for deciding when no further sampling was necessary (e.g., sampling saturation); rationale**	4/49-5/5
**Ethical issues pertaining to human subjects** - Documentation of approval by an appropriate ethics review board and participant consent, or explanation for lack thereof; other confidentiality and data security issues	13/5-11
**Data collection methods** - Types of data collected; details of data collection procedures including (as appropriate) start and stop dates of data collection and analysis, iterative process, triangulation of sources/methods, and modification of procedures in response to evolving study findings; rationale**	5/18-27

For peer review only

For peer review only - http://bmjopen.bmj.com/site/about/guidelines.xhtml

**Data collection instruments and technologies** - Description of instruments (e.g., interview guides, questionnaires) and devices (e.g., audio recorders) used for data collection; if/how the instrument(s) changed over the course of the study	5/29-47
**Units of study** - Number and relevant characteristics of participants, documents, or events included in the study; level of participation (could be reported in results)	5/60
**Data processing** - Methods for processing data prior to and during analysis, including transcription, data entry, data management and security, verification of data integrity, data coding, and anonymization/de-identification of excerpts	5/51
**Data analysis** - Process by which inferences, themes, etc., were identified and developed, including the researchers involved in data analysis; usually references a specific paradigm or approach; rationale**	5/54-56
**Techniques to enhance trustworthiness** - Techniques to enhance trustworthiness and credibility of data analysis (e.g., member checking, audit trail, triangulation); rationale**	

Results/findings

**Synthesis and interpretation** - Main findings (e.g., interpretations, inferences, and themes); might include development of a theory or model, or integration with prior research or theory	6/3-9
**Links to empirical data** - Evidence (e.g., quotes, field notes, text excerpts, photographs) to substantiate analytic findings	6/12-10/11

Discussion

**Integration with prior work, implications, transferability, and contribution(s) to the field -** Short summary of main findings; explanation of how findings and conclusions connect to, support, elaborate on, or challenge conclusions of earlier scholarship; discussion of scope of application/generalizability; identification of unique contribution(s) to scholarship in a discipline or field	10/15-12/27
**Limitations** - Trustworthiness and limitations of findings	12/30-34

Other

**Conflicts of interest** - Potential sources of influence or perceived influence on study conduct and conclusions; how these were managed	13/20-21
**Funding** - Sources of funding and other support; role of funders in data collection, interpretation, and reporting	13/25

*The authors created the SRQR by searching the literature to identify guidelines, reporting standards, and critical appraisal criteria for qualitative research; reviewing the reference lists of retrieved sources; and contacting experts to gain feedback. The SRQR aims to improve the transparency of all aspects of qualitative research by providing clear standards for reporting qualitative research.

For peer review only

**The rationale should briefly discuss the justification for choosing that theory, approach, method, or technique rather than other options available, the assumptions and limitations implicit in those choices, and how those choices influence study conclusions and transferability. As appropriate, the rationale for several items might be discussed together.

Reference:

O'Brien BC, Harris IB, Beckman TJ, Reed DA, Cook DA. **Standards for reporting qualitative research: a synthesis of recommendations.** *Academic Medicine*, Vol. 89, No. 9 / Sept 2014 DOI: 10.1097/ACM.0000000000000388

For peer review only

For peer review only - http://bmjopen.bmj.com/site/about/guidelines.xhtml

Acceptability of surgical care in Uganda: A Qualitative Study on Users and Providers

Journal:	*BMJ Open*
Manuscript ID	bmjopen-2022-070479.R1
Article Type:	Original research
Date Submitted by the Author:	03-May-2023
Complete List of Authors:	Rauschendorf, Paula; University of Bonn, Section Global Health, Institute of Hygiene and Public Health<br>Nume, Rosette; Makerere University, School of Women and Gender Studies<br>Bruchhausen, Walter; University of Bonn, Section Global Health, Institute of Hygiene and Public Health
**Primary Subject Heading**:	Global health
Secondary Subject Heading:	Qualitative research, Surgery, Health services research
Keywords:	SURGERY, Organisation of health services < HEALTH SERVICES ADMINISTRATION & MANAGEMENT, Clinical governance < HEALTH SERVICES ADMINISTRATION & MANAGEMENT, Quality in health care < HEALTH SERVICES ADMINISTRATION & MANAGEMENT

SCHOLARONE™
Manuscripts

I, the Submitting Author has the right to grant and does grant on behalf of all authors of the Work (as defined in the below author licence), an exclusive licence and/or a non-exclusive licence for contributions from authors who are: i) UK Crown employees; ii) where BMJ has agreed a CC-BY licence shall apply, and/or iii) in accordance with the terms applicable for US Federal Government officers or employees acting as part of their official duties; on a worldwide, perpetual, irrevocable, royalty-free basis to BMJ Publishing Group Ltd ("BMJ") its licensees and where the relevant Journal is co-owned by BMJ to the co-owners of the Journal, to publish the Work in this journal and any other BMJ products and to exploit all rights, as set out in our licence.

The Submitting Author accepts and understands that any supply made under these terms is made by BMJ to the Submitting Author unless you are acting as an employee on behalf of your employer or a postgraduate student of an affiliated institution which is paying any applicable article publishing charge ("APC") for Open Access articles. Where the Submitting Author wishes to make the Work available on an Open Access basis (and intends to pay the relevant APC), the terms of reuse of such Open Access shall be governed by a Creative Commons licence – details of these licences and which Creative Commons licence will apply to this Work are set out in our licence referred to above.

Other than as permitted in any relevant BMJ Author's Self Archiving Policies, I confirm this Work has not been accepted for publication elsewhere, is not being considered for publication elsewhere and does not duplicate material already published. I confirm all authors consent to publication of this Work and authorise the granting of this licence.

For peer review only

Acceptability of surgical care in Uganda: A Qualitative Study on Users and Providers

1. Corresponding author
Paula Kristina Rauschendorf
Section Global Health
Institute of Hygiene and Public Health
University of Bonn
Bonn, Germany
p.rauschendorf@outlook.de
Tel. +1(617)201-1231

2. Rosette Nume
School of Women and Gender Studies
Makerere University
Kampala, Uganda

3. Prof. Dr. med. Walter Bruchhausen Dipl.-Theol. M.Phil.(Glasg.)
Section Global Health
Institute of Hygiene and Public Health
University of Bonn
Bonn, Germany

Keywords: Health education and promotion, Surgery, Qualitative Study, Health services research

Word count: 5390

For peer review only

Acceptability of surgical care in Uganda: A qualitative study on users and providers

Abstract

Objectives This study was conducted to assess acceptability of surgical care in Eastern Uganda and enable better allocation of resources and to guide health policy towards increased surgical care seeking.

Design This qualitative study utilized semi-structured in-depth interviews that were transcribed and analysed by coding according to grounded theory.

Setting The study was set in Eastern Uganda in the districts of Jinja, Mayuge, Kamuli, Iganga, Luuka, Buikwe and Buvuma.

Participants Interviews were conducted with 32 past surgical patients, 16 community members who had not undergone surgery, 17 healthcare professionals involved in surgical treatment, and 7 District Health Officers or their deputies.

Results The five intersecting categories that emerged were health literacy, perceptions, risks and fears, search for alternatives, care/treatment and trust in healthcare workers. It was also demonstrated that considering the user and provider side at the same time is very useful for a more extensive understanding of surgical care seeking behaviour and the impact of user-provider interactions or lack thereof.

Conclusion While affordability and accessibility are well defined and therefore easier to assess, acceptability is a much less quantifiable concept. This study breaks it down into tangible concepts in the form of five categories, which provide guidance to future interventions regarding surgical care acceptability. We also demonstrated that multiple perspectives are beneficial to understanding the multifactorial nature of health care seeking and provision.

Strengths and limitations

- This study provides multiple perspectives by interviewing local health officials, (potential) users and providers of surgical care.
- Among the interviewees of each group, diversity was created through purposive sampling.
- This study is limited by its small sample size and geographical area, despite its attempt for representativity.
- Some questions needed re-phrasing after analysis of the first set of interviews following grounded theory due to them being misunderstood

For peer review only

Introduction

In 2015, the Lancet Commission Report on Global surgery highlighted the inequity of the global burden of surgical disease and access to care [1]. Surgical care, which is defined as "any intervention directed at reducing the disability or premature death associated with a surgical condition" [2], has been found to be highly effective in reducing the burden of disease and disability-adjusted life years lost [3]. Uganda faces a high unmet need for surgery [4–6], with few operating theatres (0.2 major OTs per 100,000 people) [7] and a low surgeon density of 0.73 surgeons per 100,000 persons [6]. Wards are overbooked [8] and shortages of necessities such as water, electricity, equipment, and limited access to blood banks further hinder safe surgical care [9].

The inequity in burden of surgical disease and access to care, in the context of Universal Health Coverage (UHC) as a target of the Sustainable Development Goal 3 [10] underscores the importance of *accessible*, *affordable* and *acceptable* surgical care. While accessibility [11–13] and affordability [14] of surgical care have been thoroughly researched in Uganda, acceptability of surgical care has not been a focus.

Acceptability of care was defined by Sekhon et al. as "a multi-faceted construct that reflects the extent to which people delivering or receiving a healthcare intervention consider it to be appropriate, based on anticipated or experienced cognitive and emotional responses to the intervention." [15] It is influenced by a multitude of factors such as the quality of healthcare facilities, availability and attitudes of and towards healthcare personnel, perceptions, risks and fears associated with healthcare, available alternatives and health literacy, which can be defined as "the degree to which individuals have the ability to find, understand, and use information and services to inform health-related decisions and actions for themselves and others." [16]

Research on acceptability of care can provide insights into reasons for low uptake of surgical care and inform resource allocation and health policies. Acceptability of care needs to be assessed from multiple perspectives: patients, as well as the broader public, health care providers and health officials can each provide a unique viewpoint. Therefore, this study analyzes interviews with former surgical patients, community members, surgical healthcare professionals, and District Health Officers.

Methods

Study design

This qualitative study utilized semi-structured in-depth interviews that were transcribed, if needed translated and analysed by coding according to grounded theory. Grounded theory is a qualitative research method aimed at developing theories from data and their analysis [17–19] that can increase understanding of social behaviours and processes, e.g. in patient care [20]. Due to its inductive nature, it is often used in fields with little prior research.

Patient and public involvement

There was no patient or public involvement in the study prior to data collection.

Setting and participants

The interviews were conducted from August to September 2019 in the districts of Jinja, Mayuge, Kamuli, Iganga, Luuka, Buikwe and Buvuma. They are located in South-Eastern Uganda and have an estimated population of 2.84 million [21]. The Eastern region of Uganda has a significantly higher poverty rate than the national average. [22]

The goal was to interview members of four groups in each district:

For peer review only

- six former surgical patients (FP) and
- three community members who had not had surgery (CM) from three different communities, which were selected purposively with regards to distance to the next surgery providing health care facility and distance from the main road. One community was chosen near a surgical facility, one was chosen to be further from a facility but along the main road and one was chosen further away from both the facility and the main road.
- three healthcare workers involved in surgical care (HW), including representatives from a private for profit (PFP), private non-for-profit (PNFP) and government (public) facility. In districts where PFP or PNFP facilities were lacking, either two interviews were conducted at a government facility, or one interview was conducted in the district. To ensure diversity and minimize selection bias, different medical cadres were interviewed in each sector, following purposive sampling.
- the District Health Officers (DHO).

Figure 1

Interview guide

The process of developing the interview guides involved reviewing relevant literature, identifying key concepts or themes, and crafting open-ended questions to elicit in-depth responses from the interviewees. The final interview guides were structured in a logical sequence to facilitate a smooth flow of the interviews and to ensure that all relevant topics were covered. The interview guides served as a flexible tool that allowed for probing and follow-up questions based on the responses of the interviewees, ensuring that a rich and comprehensive data set was collected during the interviews. After analysis of the first set of interviews, some questions were rephrased for clarity.

During the interviews, FPs were asked about their experience with surgery, delays in healthcare seeking, reasons for uncertainty, consultations outside of biomedical healthcare, decision making, the process of their surgical treatment, changes in surgery perception, community support and suggestions for improvement of health facilities. CMs were asked about their thoughts on surgery and their perceived struggles, health behaviours, knowledge on surgical conditions, financial impact, health insurance coverage and their concerns pertaining to surgery.

HWs were asked about their experience in providing surgical care, difficulties, improvements, and patient perceptions. Interviews with DHOs commenced with a question about obstacles in providing access to healthcare, particularly surgical care, and followed by questions about supply chains, health education campaigns, the surgical workforce and ways of involvement of NGOs.

For full interview guides please refer to Appendix 1-4.

Data collection

Overall, 72 interviews were conducted. Community interviews were conducted in Lusoga. Interviews with HWs and DHOs were conducted in English or Lusoga depending on preference.

The DHO was approached for an interview and simultaneously permission was obtained to interview HWs in the district. Then HWs were invited to participate in the study at their respective healthcare facilities. Community members were engaged and asked if they had undergone surgery or could refer to someone who had. This process was repeated until two FPs and one CM were recruited.

Theoretical saturation was reached towards the end of sampling, meaning that new information or insights were no longer emerging from the data. This suggests that the sample size was sufficient for addressing the research questions and achieving the research objectives.

For peer review only

Interviews were audio recorded, transcribed verbatim and anonymized. Translations to English were performed if the original language of the interview was not English.

Data analysis

Data was analysed using MAXQDA Pro Analytics. MAXQDA is a qualitative data analysis software that provides various tools and features for analysing qualitative data. The software allows researchers to organize, code, and analyse text, audio, video, and image data in a systematic and rigorous manner. Interview transcripts were coded according to grounded theory by finding the key words in each section of the interviews. Codes were grouped into sub-categories and sorted into larger categories.

Results

Interviews were conducted with 33 FP, 16 CM, 17 HW and 7 DHO or their deputies.

Demographic data of the sample can be found in Table 1 and 2.

For peer review only

Table 1. Demographic characteristics of FP and CM

	Former Patients		Community members	
**Characteristic**		**Percentage**		**Percentage**
**Sex**				
Female	21	63.6	10	62.5
Male	12	36.4	6	37.5
**Age (years)**				
<20	3	9.1	0	0.0
21-40	12	36.4	3	18.8
41-60	12	36.4	10	62.5
61-80	5	15.2	3	18.8
>80	1	3.0	0	0.0
**Level of education**				
Tertiary	3	9.1	2	12.5
Secondary	8	24.2	3	18.8
Primary	9	27.3	11	68.8
None	5	15.2	0	0.0
No answer	8	24.2	0	0.0
**Employment status**				
Self-employed	2	6.1	4	25.0
Unemployed	16	48.5	11	68.8
Formal employment	6	18.2	0	0.0
Retired	1	3.0	0	0.0
No answer	8	24.2	1	6.3
**Time of surgery**				
2015-2019	15	45.5		
2010-2014	4	12.1		
2005-2009	3	9.1		
Before 2005	1	3.0		
No answer	10	30.3		
**Facility type**				
Public	16	48.5		
PNFP	5	15.2		
PFP	8	24.2		
No answer	4	12.1		

For peer review only

Table 2. Demographic characteristics of HW

	Healthcare workers	
**Characteristic**		**Percentage**
**Sex**		
Female	4	23.5
Male	13	76.5
**Profession**		
Nurse	1	5.9
Midwife	1	5.9
Theatre assistant	1	5.9
Medical officer	4	23.5
Medical doctor	6	35.3
Surgeon	3	17.6
Obstetrician	1	5.9
**Facility type**		
Public	8	47.1
PNFP	5	29.4
PFP	4	23.5

During the analysis, five dimensions of acceptability of surgical care emerged. *Health literacy* was found to impact healthcare seeking behaviour and shape expectations about surgical care, which were further influenced by *fears and perceptions of surgery*. Comparisons were made between surgical care and *alternative healthcare options*. Trust in the *healthcare worker* as well as *care and treatment* emerged as important categories influencing acceptability.

Health literacy: impact and improvement

Low levels of health literacy impacted healthcare seeking negatively according to many participants from all groups. A DHO lamented that patients did not know the "*timely need for coming for healthcare*". HWs supported this and explained that "*people don't know that they require surgery*" (surgeon, PNFP) or "*the impact of their condition*" (medical doctor (MD), PNFP). An FP mentioned that a mass in his spleen was discovered during an intestinal obstruction surgery one year before, but he did not seek further medical evaluation afterward.

Many FPs reported waiting for a long time before seeking care, as one said: "*I delayed a bit. I think it was around [...] 10 years*" (FP, PFP). Along with other participants, another FP attributed the delay to his "*ignorance, because I wasn't aware the illness needed a medical treatment*" (FP). Some participants described self-medicating, as in this example: "*When I am sick, I just buy medicine of 2000 to 3000 shillings and if that sickness doesn't respond to the treatment, I continue changing the type of treatment until I get better.*" (CM)

Low levels of general education were discussed, since "*perceptions are about education*" (medical officer (MO), public). A HW described struggling to find a comprehensible way to explain surgical conditions to the patients. CMs similarly said: "*after taking blood samples from us, there's nothing you can understand*" (CM) and "*those who never went to school don't know anything and you have to believe all what the healthcare workers tell you*" (CM).

While many CMs demonstrated knowledge on obstetric conditions and hernia, other conditions such as goitre, ulcer, and cancer were less frequently mentioned. A HW attributed this to the fact that

For peer review only

"*surgery doesn't have enough advocacy, but obstetric, emergency obstetric care has received a lot of advocacy and a lot of funding*" (MO, public). He described the increase in health education campaigns around obstetric emergencies had led to women "*coming to the facilities, seeking care, surgical care compared to general surgery*." (MO, public). Another MO (PNFP) confirmed the importance of such campaigns for recruiting patients.

Therefore, more health education specific to surgery was proposed to improve surgical care seeking. Suggestions included campaigns broadcasted via radio and televisions as well as outreaches and village health teams (VHTs) "*to identify some of these complications – the illnesses – and educate, give some health education and refer these people to where they can get services*" (Surgeon, PFP). One HW raised concerns that educating the population had to be linked with building surgical capacity to accommodate the anticipated increase in surgical patients.

Perceptions and Fears of Surgery

Surgery was perceived by CMs and FPs as a way of "*reducing the sickness that is bothering you*" (FP, PNFP) by "*cutting you open to see the cause of sickness and remove the unwanted stuff*" (FP, PFP), oftentimes with urgency: "*the patient is in a critical and not understandable condition, then why can that patient not be taken to be operated*" (CM). Surgical care was described as a last resort by CMs and FPs, often only an option "*when the condition that calls for the operation is very extremely severe, when the pain is so so so beyond, without giving you time to eat, drink or walk, I mean when the illness is at its maximum, then there you can decide to go for the operation*" (CM).

The risk associated with surgery was often named as "*you can come back or not come back*" (CM). A surgeon (PNFP) attributed these sceptical attitudes to the fact that "*in the past the results from surgery have not been good. It has been between life and death*." One of the DHOs mentioned that cases with complications are overly emphasised causing a prevalent misconception that surgery is unsafe. Seeming medical malpractice in anaesthesia was described by one FP (PFP): "*When I was still half sedated, they cut me but the way they pulled my scrotum with much force I felt terrible pain in the intestines*".

Participants mentioned the fear that surgeons would leave instruments or swabs in the patient with possible complications and re-operations. One recounted that the people "*normally say that when they go for surgery, they make you more disabled not allowed to do certain work*" (CM). Another example was the fear that putting a patient "*on oxygen means he's going to die*" (nurse, PNFP). Also, there were "*rumours that healthcare workers steal certain organs from the patients*" (CM) which led to fear among the community. One FP (public) suggested having a relative of the patient in the theatre to "*see the procedures of the operation and what they have removed from the patient*".

One midwife (PFP) saw an increase in trust after successful operations, which was also apparent in some interviews with FPs: "*healthcare providers can operate and after the operation you can wake up and resume with normal walking, healthy person, and the illness is taken away you can become better, and you live a healthy life.*" (FP, PNFP) Another FP (PFP) described how she was asked after her surgery "*not to scare my fellow women that the health facility was a terrible place; they told me to advise my friends to go for medical procedures*". This demonstrates that staff feels the need for improving the reputation of surgical treatment. As a way of counteracting patient fear, effective pre-operative counselling was suggested by FPs and healthcare providers.

Understanding of Illness and the Search for Alternatives

Surgical conditions as seen from a biomedical perspective were mostly regarded by FPs and CMs as necessitating biomedical treatment, and many participants named biomedical facilities as their only source of healthcare. Patients mostly became aware that their condition was surgical after it had

For peer review only

been identified as such by a healthcare worker. One FP (PNFP) stated: "*sickness that is meant for operation, still goes for operation, there is no alternative*". In contrast to the view that surgical conditions always necessitate surgery, one medical officer described that pertaining to surgical diagnosis "*they [the patients] think there is something that you have not done. They think you should have given some medication for that condition*."

Herbal medicine, for example was described as a way of preventing surgery under certain circumstances: "*I was to have a normal delivery if I used the local herbs*" (FP, public). It was only mentioned in the context of chronic conditions like hernia, not as an alternative in emergency or trauma cases. An FP described "*someone went to those healers and was given herbs and his hernia disappeared, so I also went there and used those herbs for one day but when I noticed no improvement, I gave up on them*." (FP, PFP). Another example was given by an FP (PFP) with haemorrhoids, who was later operated: "*when I applied the herbs, the piles went back but after some time, it re-occurred, and when I asked them about that, they told me that, the piles can only be healed with traditional medicine and so I continued with that medicine.*" HWs expressed frustration but blamed it on the "*traditional healers or herbalists who keep misguiding or giving false information to our clients*" (surgeon, PFP) and the lack of knowledge concerning surgical conditions.

Similarly, when asked about traditional healers in the area, many respondents voiced distrust because they would not "*reveal that your condition needs surgical treatment. When they want money, they just divert you from seeking medical care, yet they can't treat the problem at hand.*" (CM) However, some participants also saw fault with the patients that sometimes "*cannot differentiate a medical problem from a traditional one*" (CM) and "*think that they have been bewitched, even if the sickness is not traditional*" (FP). This shows an understanding of ill-health and healing of two separate spheres with different illnesses and their corresponding healthcare, i.e. an alternative between "medical" and "traditional".

Differing from this understanding of two completely distinct types of illness and treatment, a CM argued "*those other things of bewitching can come later after going to the health facility*" (CM). Here the common view of complementary functions is expressed that biomedicine may – even quickly – remove the damage like hernia, but not the underlying cause like bewitchment, as one said: "*Can a healthcare worker tell you issues of the home? […] But it is the traditional healer to tell you the facts. The healthcare worker can suspect the cause of illness and tell the patient that maybe the patient delayed getting treatment, but when you go to these ones, they can tell you.*" (CM).

Care and Treatment

The received care was commonly described as good. Good was, however, defined differently by FPs, e.g. as not being mistreated or being spoken to "*in a friendly manner*" (FP, PNFP). Others voiced more specific expectations: "*to be cared for, is when the healthcare worker comes in the ward and greets you asking about how the night was, how you feel, the condition of the baby and other issues like that.*" (FP, public) For others the focus was more on the medical aspects like receiving "*timely treatment*" (FP, PFP).

The type of care received was commonly attributed to the patient's financial situation, where paying the asked amount was linked to good care and medical treatment, whereas patients with financial constraints were often mentioned to receive worse treatment. Other FPs attributed the good care they received to personal relationships with the healthcare workers. One FP (PNFP) reported that "*they respected me because first of all I am educated, and I interacted with them in English during all the chats*".

For peer review only

Despite general satisfaction, some complaints were voiced like going through episodes of extreme pain after surgery, some to the extent, that it would prevent future surgical care seeking. Other FPs mentioned mistreatment by healthcare workers, for example, a female FP (public) was on a trolley to the operation theatre for a C-section when she "*felt something like a baby coming out then I widened my legs as if I was going to deliver, then the nurse slapped me for widening the legs, and exposing my nakedness to the people*". The nurse was held accountable for her actions. A CM lamented: "*if you are lucky enough and your patient is operated, they carelessly stich and just dump him/her there for you. They are always not concerned with the aftereffects of the operation, it's you to care for the patient.*"

This alludes to the important role of informal caregivers in Ugandan healthcare. "*They carry the burden, the financial, emotional, and so on*" (surgeon, public). Their importance was highlighted multiple times throughout interviews, for example when one FP (public) said, "*if you have no effective caregiver, it's a big challenge*". The informal caregiver has different responsibilities, mainly taking care of the basic needs of patients, while at the hospital and to provide emotional support. One FP (public) stated, that when patients did not have their treatment readily prepared by their informal caregiver nurses "*just pass to another patient*".

The "*lack of proper trained surgical nursing care*" (medical superintendent) led to some surgeons feeling "*let down by the postoperative care*" (MO, public). A nurse (PNFP) explained: "*we are like 2 or 3 on duty and patients are many and we have to attend to them, sometimes you find that you have not reached up to that peak of caring for the patient*". Therefore, sometimes "*responsibilities channel to the relatives*" (MO, public) that should be carried out by medical personnel. A surgeon noted that this led to problems in the aftercare of operations. HWs also noted that the lack of separate surgical wards was a challenge to providing specialised care. To counteract these challenges, more training, a separate surgical ward and an increase in staff were suggested.

Follow-up of operations was another theme that emerged. Some FPs said they had not been asked back for follow-up. HWs commonly said that review dates were scheduled with patients, but attendance varied. This was a challenge for surgical staff, as doctors were then "*not able to monitor them for any complications*" (Surgeon, PFP). Varying commitment from HWs was apparent, with some recording patients' numbers and calling to check up and some visiting their patients at home. A surgeon (PFP) suggested that regular "*field visits*" could improve follow-up.

Healthcare workers: Trust and Misbehaviour

Closely linked to care and treatment was the patient-healthcare worker relationship. As one CM stated: "*the healthcare worker is the overall in this thing, he/she is the key to everything*". Trust and lack thereof were commonly mentioned.

Most interviewed HWs agreed that they felt trusted by patients. Some FPs described a trustful relationship, with one mentioning being taken care of by a doctor off-duty and another calling the doctor a "*saviour*" (FP, public). One medical officer (public) differentiated: "*Those who come, trust us. What they don't trust is the system. What they don't trust is their government*". Contrastingly, distrust in the surgical skills of healthcare workers was also mentioned with one FP saying: "*most of the healthcare workers are young boys, we don't trust them*". This is caused by the regulation that demands service in remote communities in the first years after graduation. One doctor (PFP) highlighted that this was a major difference to traditional healers, as they "*tend to give more time to their patients actually and convince them adequately*". The overwhelming workload and little time spent with the patient were given as reasons for the lack of trust in the healthcare system and HWs.

For peer review only

HWs were expected to "*first think about people's health, then think about money*" (CM) because "*there are rewards in heaven*" (CM). Work ethics of HWs were a concern to participants from the community, who described nurses and doctors commonly asked for informal payments. One FP (public) described being asked "*to pay her 60,000 shillings for quick treatment and we go home instead of being admitted in the ward*"). Some mentioned, HWs would let the patient die if they could not pay: "*There was that first one I saw, he had even refused to operate me, and if the second one never showed up, I was going to die*" (FP, public). Participants suggested to raise salaries of HWs, so they would stop demanding informal payments.

Medical personnel generally described their own work ethics as high, with one medical officer calling it a "*commitment to serve*". One doctor (PNFP) described having to pay for medical equipment when the patient cannot afford it: "*sometimes it is necessary, where you see that life is more important*". Nevertheless, many saw issues with motivation and work morale in the health workforce in general, attributing it to frustration due to poor salaries, long working hours, poorly equipped facilities, and little opportunity for continued education and mentorship. Scholarships for further education and mentorship were mentioned as important. Training programmes through foreign exchange or as part of surgical camps were seen as beneficial for both sides: "*Probably you might come with the skills where you come from, but the context differs. Then you also have something to learn with us, from us working with limited resources.*" (DHO)

Discussion

In providing surgical care to communities, like in Uganda, factors beyond accessibility and affordability necessitate careful consideration. While some commonly used models like the Behavioural Model of Health Service Use by Andersen [23] consider mostly the user side, this study aims to showcase the user, the provider and the health officials' perspective. The five areas found to impact acceptability of surgical care were: health literacy, perceptions of surgery, the search for alternatives, care and treatment and the relationship to the healthcare worker. These categories, however, had some overlap.

Higher health literacy has been shown to increase acceptance of surgical care [24] while limited health literacy has been linked to worse outcomes and lower satisfaction [25] and to overall non-adherence to pre- and postoperative instructions [26]. Participants of our study, especially on the provider side, also viewed health literacy on surgical conditions impactful for improving surgical care seeking. It should be a focus of future interventions, as suggested by Butler et al. in a study where lack of perceived need left 25% of children with surgical needs untreated [27]. Health literacy assessments have been shown to be feasible and to enable increased patient satisfaction [28]. General education and literacy were also discussed by participants as enabling patients to make better decisions on healthcare seeking for surgical conditions, which was also found by Fuller et al. and Davé et. al [29].

A thorough understanding of the perceptions of surgery and concepts of illness is necessary to provide acceptable care. The question of what is at stake for those people most concerned by a surgical condition needs to be addressed for meeting important expectations and creating trust. Understandably sceptical attitudes due to former negative experiences with formal education and biomedical healthcare [30] as well as unfavourable surgical outcomes [31] have a large impact on surgical healthcare seeking.

While the surgical treatment was often described as successful by patients, negative perceptions were commonly mentioned. Risks and fears related to surgical interventions act as a barrier for their uptake. Perceptions were also shaped by rumours, like concerns of organ stealing. Rumours like this

For peer review only

have existed in many African societies and have been discussed extensively by White [32] and Scheper-Hughes [33]. Other rumours noted were specific to surgical care and to proceedings of an operating room. Improving health literacy, setting a focus on communication with patients and improved education prior to surgery could play an important part in counteracting rumours. It could also reduce self-medication and change the current role of surgery as a last resort or for emergencies only.

With 4.0% of rural children with surgical conditions not receiving care due to fear [34], trust and positive interactions with surgical care providers are important. While most healthcare personnel felt trusted by patients and many patients voiced trust, some scepticism towards surgical skills particularly of medical officers was expressed. Similar concerns about seniority and skill level were voiced by participants in a study by Mwaka et al suggesting that only senior and skilled doctors were regarded as fit to successfully operate cancer patients [35]. Due to lack of specialists, non-specialised personnel commonly conducts surgery in Uganda [36].

Participants in our study, especially medical officers, wished for more mentorship and further specialisation. This would also provide an opportunity for more training on interpersonal skills to improve communication with patients, as suggested by Bohren et. al [37]. Surgical camps were suggested as possible training opportunities. Modern technology was also proposed to improve mentorship, where medical officers in remote areas would be able to consult with colleagues in Uganda or abroad via telecommunication.

Most FPs and CMs recognized the need for operative care in case of surgical conditions. These findings align with results from a previous study investigating surgery beliefs of lay people, where surgery was seen as “the appropriate healing method”, although there was also “fear of negative outcome of surgery” [35]. In our study, some participants mentioned attempting alternative treatments under the category of “traditional medicine”, which was also a common theme among providers. Participants from communities often agreed there were indications for traditional medicine, however rarely including surgical conditions like hernia.

Many participants from all groups highlighted the dangers of misdiagnosis and delay of treatment, which has been previously reported [5, 27] and was discussed by WHO in their traditional medicine strategy [38]. However, patients continue to seek care from traditional healers, which healthcare providers attributed to the fact that traditional healers often spend more time with patients. This could be due to the ratio of 1 traditional healer per population of 500, compared to 1 medical doctor per 25000 people in Uganda [39]. Participants also discussed their role as holistic healers. Some of these features could possibly be a starting point for developing a healthcare system encompassing the underlying needs of patients answered by traditional medicine.

Overall, patients in this study were satisfied with care, but noted that factors such as financial situation, education level and relationships with healthcare workers influenced the type of care received. Similar findings were reported in other studies, citing bribes, socio-economic class, political affiliation, and tribal favouritism [40] as well as social capital and financial leveraging [41] as factors impacting care. Participants in our study linked these behaviours to the work ethics of HWs. While HWs expressed high motivation, many described low morale among the health workforce, due to factors such as low salaries, difficult work environments and lack of mentorship.

Participants described negative interactions between healthcare providers and patients, including harsh treatment and neglect. This is consistent with previous findings from Benin [31]. Kagoya et al. found a lack of negotiating power among patients at the Ugandan National Referral Hospital caused by “illiteracy, language barriers, low socio-economic status, feeling inferior to HWs and perceived favour to access free services” [40].

For peer review only - http://bmjopen.bmj.com/site/about/guidelines.xhtml

Some FPs described situations which could be interpreted as malpractice or errors in surgical care. A study on general medical errors found how they occurred and that healthcare professionals were mostly aware of them [42]. In regards to surgical errors, “inappropriate pre-operative management, omitting preoperative investigations and omitting post-operative notes“ [43] were most commonly found by reviewing patient records. A lack of error reporting on medication errors was also found by Kiguba et. al. [44].

Post-surgical care and follow-up were seen as major challenges by patients and healthcare providers. Lack of trained nursing staff leads to task shifting to informal caregivers, as highlighted by participants. In post-operative caregiving, informal caregivers often lack the necessary training, education and understanding of medical conditions necessary to provide the best care possible. Formerly proposed caregiver training to increase capacity of post-surgical care [45] was not suggested by participants in this study, who laid more focus on increasing the surgical workforce.

Previous research suggests that receiving follow-up care is strongly dependent on socio-economic status and distance to access points [45]. Our findings support this. Healthcare professionals in our study suggested taking phone numbers of patients and making fixed appointments to combat this problem. Home visits were mentioned as ideal, but not feasible. A study on maternal and new-born care practices also suggests that a combination of home visits by VHTs and mobile phone consultations improve care [46]. Interventions linked to VHTs should be strengthened and possibly expanded to include regular basic health check-ups to facilitate earlier interventions on complications and remissions.

Limitations

In its aim to provide a broad overview of perspectives on surgical care, this study is limited by its small sample size and geographical area, despite its attempt for representativity. However, the broader categories that emerged may be applicable to the country and could even find application beyond. The study also showed that grounded theory can be applied to study acceptability of care in specific contexts.

In the first interviews participants were not asked to provide the year of their surgery. In the selection of participants, no exclusion criteria were defined regarding the year of surgery. While this approach may have limited representativity for current surgical care, it was intentional as it was believed that experiences from the past may still shape perceptions and attitudes within the communities, even if the surgery occurred long ago.

The educational, social science, medical, and cultural backgrounds of the researchers conducting the interviews, being a Ugandan graduate student and a German medical doctoral student, may have created a sense of authority or power dynamic that could have impacted the participants' answers. This introduces the potential for observer bias in the study, as the presence of the researchers during the interviews could have influenced the responses of the participants.

Concerning both, reliability and validity, some questions needed re-phrasing after grounded theory analysis of the first set of interviews since they had been misunderstood.

Conclusion

This study emphasises the need to consider the views of users, providers and local health officials for a comprehensive understanding of factors influencing the acceptability of surgical care. Expectations of surgical care emerged to be shaped by *health literacy* and *perceptions* of illness and surgery. Extensive information and health education campaigns on surgical conditions could improve healthcare seeking and empowerment of patients and their relatives. Reasons for seeking out

For peer review only

alternative treatments can provide insights for future interventions. The *care and treatment* at large as well as the *relationship with the healthcare worker* impacted acceptability. Negative experiences by patients could be addressed by improving the training of medical personnel, enhancing interpersonal skills and promoting a respectful environment in hospitals and healthcare settings. The needs of providers should be taken into consideration to improve motivation and workplace satisfaction. Structural improvements and an increase in qualified staff are the basis for a work environment in which acceptability can be given the necessary attention. Finally, our findings suggest the need for more extensive research on the underlying mechanisms of acceptability of surgical care, as an important foundation to tackle delays in surgical care seeking.

Ethical considerations

Permission to conduct the interviews was obtained from the Ugandan National Council for Science and Technology after receiving ethical clearance from Mildmay Uganda Research Ethics Committee under #REC REF 0102-2019. For interviews with DHOs permission was given by the Ministry of Health. Furthermore, we asked permission of DHOs to conduct interviews with HW in their district. All participants provided informed consent to be eligible to participate in this study.

Data Sharing statement

We provided all relevant excerpts within the manuscript as raw data cannot be shared publicly due to the significant and valid confidentiality concerns of interviewees, small sample size, and ethics approval requirements.

Conflict of Interest

The author(s) certify that they have no conflict of interest in the subject matter or materials discussed in this manuscript.

Funding

The study was funded by the Section Global Health at the University Hospital Bonn.

Author Contributions

PR, a German medical doctoral student at the time of the study, and WB, a professor for Global Health at the Universities of Cologne and later Bonn, developed the study design and the interview guides. Data collection was carried out collectively by PR and RN, a master's student at the School of Women and Gender Studies. PR then conducted the data analysis and prepared the manuscript. WB oversaw all aspects of this research. All authors contributed to the article and approved the submitted version.

Acknowledgements

We thank Global Health at University Hospital Bonn for the financial support, Ando-Modular aid e.V. for the inspiration for this study, Hatiika Namyalo for helping us navigate the Ugandan health care system and Grace Meara for helpful discussions.

For peer review only

REFERENCES

1 Meara JG, Leather AJM, Hagander L, et al. Global Surgery 2030: evidence and solutions for achieving health, welfare, and economic development. *Lancet* 2015;386(9993):569–624. http://www.sciencedirect.com/science/article/pii/S014067361560160X.

2 Bickler S, Ozgediz D, Gosselin R, et al. Key concepts for estimating the burden of surgical conditions and the unmet need for surgical care. *World J Surg* 2010;34(3):374–80.

3 WHO Executive Board. Strengthening emergency and essential surgical care and anaesthesia as a component of universal health coverage: Report by the Secretariat 2014.

4 Butler EK, Tran TM, Nagarajan N, et al. Epidemiology of pediatric surgical needs in low-income countries. *PloS one* 2017;12(3):1-9. doi:10.1371/journal.pone.0170968 [published Online First: 4 March 2017].

5 Tran TM, Fuller AT, Butler EK, et al. Surgical need among the ageing population of Uganda. *Afr Health Sci* 2019;19(1):1778–88.

6 Farber SH, Vissoci JRN, Tran TM, et al. Geospatial Analysis of Unmet Surgical Need in Uganda: An Analysis of SOSAS Survey Data. *World J Surg* 2017;41(2):353–63.

7 Linden AF, Sekidde FS, Galukande M, et al. Challenges of surgery in developing countries: a survey of surgical and anesthesia capacity in Uganda's public hospitals. *World journal of surgery* 2012;36(5):1056–65.

8 Nwanna-Nzewunwa OC, Ajiko MM, Kirya F, et al. Barriers and facilitators of surgical care in rural Uganda: a mixed methods study. *The Journal of surgical research* 2016;204(1):242–50. doi:10.1016/j.jss.2016.04.051 [published Online First: 28 July 2016].

9 Albutt K, Yorlets RR, Punchak M, et al. You pray to your God: A qualitative analysis of challenges in the provision of safe, timely, and affordable surgical care in Uganda. *PloS one* 2018;13(4):1-16. doi:10.1371/journal.pone.0195986 [published Online First: 18 April 2018].

10 UN General Assembly. Transforming our World: The 2030 Agenda for Sustainable Development 2015.

11 Albutt K, Namanya D, Anderson G, Kayima P. Uganda. In: Johnson W, Lin Y, Mukhopadhyay S, Meara J, ed. Surgical care systems strengthening: developing national surgical, obstetric and anaesthesia plans. Geneva: World Health Organization 2017:4–7.

12 Albutt K, Drevin G, Yorlets RR, et al. 'We are all serving the same Ugandans': A nationwide mixed-methods evaluation of private sector surgical capacity in Uganda. *PLoS One* 2019;14(10):e0224215.

13 Albutt K, Punchak M, Kayima P, et al. Access to Safe, Timely, and Affordable Surgical Care in Uganda: A Stratified Randomized Evaluation of Nationwide Public Sector Surgical Capacity and Core Surgical Indicators. *World J Surg* 2018;42(8):2303–13.

14 Anderson GA, Ilcisin L, Kayima P, et al. Out-of-pocket payment for surgery in Uganda: The rate of impoverishing and catastrophic expenditure at a government hospital. *PloS one* 2017;12(10):1-13.

15 Sekhon M, Cartwright M, Francis JJ. Acceptability of healthcare interventions: an overview of reviews and development of a theoretical framework. *BMC Health Serv Res* 2017;17(1):88. doi:10.1186/s12913-017-2031-8 [published Online First: 26 January 2017].

16 U.S. Department of Health and Human Services. Health Literacy in Healthy People 2030 - Healthy People 2030 | health.gov 2023. Available at: https://health.gov/healthypeople/priority-areas/health-literacy-healthy-people-2030 Accessed April 22, 2023.

17 BG Glaser. The discovery of grounded theory: Strategies for qualitative research: Glaser BG, Strauss AL. The discovery of grounded theory: Strategies for qualitative research. USA: Transaction Publishers; 2009 2009.

For peer review only

18 J Corbin. Basics of qualitative research: Techniques and procedures for developing grounded theory: Corbin J, Strauss A. Basics of qualitative research: Techniques and procedures for developing grounded theory. Thousand Oaks: Sage publications; 2014 2014.
19 K Charmaz. Constructing grounded theory: Charmaz K. Constructing grounded theory. London: Sage publications; 2014 2014.
20 Higginbottom G, Lauridsen EI. The roots and development of constructivist grounded theory. *Nurse Researcher* 2014;21(5):8–13.
21 Uganda Bureau of Statistics. Projected population by district (2015-2030) 2019.
22 Uganda Bureau of Statistics, UNICEF, World Bank. Poverty Maps of Uganda: Mapping the Spatial Distribution of Poor Households and Child Poverty Based on Data from the 2016/17 Uganda National Household Survey and the 2014 National Housing and Population Census. Technical Report 2019.
23 Andersen R, Davidson P. Improving access to care in America: Individual and contextual indicators. *Changing the US Health Care System: Key Issues in Health Services Policy and Management* 2014;2007.
24 Biyik I, Usturali Mut AN, Albayrak M, et al. Effect of health literacy on help-seeking behavior: A comparison of patients accepting surgery and refusing surgery for urinary incontinence. *J Gynecol Obstet Hum Reprod* 2021;50(4):101908.
25 Roh YH, Lee BK, Park MH, et al. Effects of health literacy on treatment outcome and satisfaction in patients with mallet finger injury. *J Hand Ther* 2016;29(4):459–64. doi:10.1016/j.jht.2016.06.004 [published Online First: 17 October 2016].
26 Oliveira GS de, McCarthy RJ, Wolf MS, et al. The impact of health literacy in the care of surgical patients: a qualitative systematic review. *BMC Surg* 2015;15:86. doi:10.1186/s12893-015-0073-6 [published Online First: 17 July 2015].
27 Butler EK, Tran TM, Fuller AT, et al. Quantifying the pediatric surgical need in Uganda: results of a nationwide cross-sectional, household survey. *Pediatric surgery international* 2016;32(11):1075–85. doi:10.1007/s00383-016-3957-3 [published Online First: 12 September 2016].
28 Komenaka IK, Nodora JN, Machado L, et al. Health literacy assessment and patient satisfaction in surgical practice. *Surgery* 2014;155(3):374–83. doi:10.1016/j.surg.2013.10.011 [published Online First: 14 October 2013].
29 Davé DR, Nagarjan N, Canner JK, et al. Global Burden of Craniofacial Disorders: Where Should Volunteering Plastic Surgeons and Governments Focus Their Care? *J Craniofac Surg* 2020;31(1):121–24.
30 Raykar NP, Yorlets RR, Liu C, et al. The How Project: understanding contextual challenges to global surgical care provision in low-resource settings. *BMJ Global Health* 2016;1(4):e000075. doi:10.1136/bmjgh-2016-000075 [published Online First: 16 December 2016].
31 Close KL, Christie-de Jong FTE. Lasting impact: a qualitative study of perspectives on surgery by adult recipients of free mission-based surgical care in Benin. *BMJ Open* 2019;9(11):e028235. doi:10.1136/bmjopen-2018-028235 [published Online First: 7 November 2019].
32 White L. Speaking With Vampires: Rumor and History in Colonial Africa. Berkeley, Calif.: University of California Press 2000.
33 Scheper-Hughes N. Parts unknown. *Ethnography* 2004;5(1):29–73.
34 Bearden A, Fuller AT, Butler EK, et al. Rural and urban differences in treatment status among children with surgical conditions in Uganda. *PLoS One* 2018;13(11):e0205132.
35 Mwaka AD, Okello ES, Wabinga H. Perceptions and beliefs of lay people from northern Uganda regarding surgery for diagnosis and treatment of cervical cancer. *Psychooncology* 2018;27(8):1965–70.

For peer review only

36 Baine SO, Kasangaki A, Baine EMM. Task shifting in health service delivery from a decision and policy makers' perspective: a case of Uganda. *Hum Resour Health* 2018;16(1):1–20. doi:10.1186/s12960-018-0282-z [published Online First: 2 May 2018].

37 Bohren MA, Titiloye MA, Kyaddondo D, et al. Defining quality of care during childbirth from the perspectives of Nigerian and Ugandan women: A qualitative study. *Int J Gynaecol Obstet* 2017;139 Suppl 1:4–16. doi:10.1002/ijgo.12378 [published Online First: 7 December 2017].

38 World Health Organization. WHO traditional medicine strategy 2014 - 2023. Genève: WHO 2013.

39 Abdullahi AA. Trends and challenges of traditional medicine in Africa. *Afr J Tradit Complement Altern Med* 2011;8(5 Suppl):115–23. doi:10.4314/ajtcam.v8i5S.5 [published Online First: 3 July 2011].

40 Kagoya HR, Kibuule D, Mitonga-Kabwebwe H, et al. Awareness of, responsiveness to and practice of patients' rights at Uganda's national referral hospital. *Afr J Prim Health Care Fam Med* 2013;5(1).

41 Stephens T, Mezei A, O'Hara NN, et al. When Surgical Resources are Severely Constrained, Who Receives Care? Determinants of Access to Orthopaedic Trauma Surgery in Uganda. *World J Surg* 2017;41(6):1415–19. https://pubmed.ncbi.nlm.nih.gov/28097413/.

42 Mauti G, Githae M. Medical error reporting among physicians and nurses in Uganda. *Afr Health Sci* 2019;19(4):3107–17. https://pubmed.ncbi.nlm.nih.gov/32127887/.

43 Katongole Simon Peter, Robert Anguyo, Ddm Onzima, et al. Common Medical Errors and Error Reporting Systems in Selected Hospitals of Central Uganda 2015.

44 Kiguba R, Waako P, Ndagije HB, et al. Medication Error Disclosure and Attitudes to Reporting by Healthcare Professionals in a Sub-Saharan African Setting: A Survey in Uganda. *Drugs Real World Outcomes* 2015;2(3):273–87.

45 Barton SJ, Sandhu S, Doan I, et al. Perceived barriers and supports to accessing community-based services for Uganda's pediatric post-surgical population. *Disabil Rehabil* 2019:1–12.

46 Mangwi Ayiasi R, Kolsteren P, Batwala V, et al. Effect of Village Health Team Home Visits and Mobile Phone Consultations on Maternal and Newborn Care Practices in Masindi and Kiryandongo, Uganda: A Community-Intervention Trial. *PLoS One* 2016;11(4):e0153051. doi:10.1371/journal.pone.0153051 [published Online First: 21 April 2016].

Figure Legend

Figure 1. Sampling strategy per district. The goal was to conduct 6 interviews with FPs, 3 interiews with CMs from 3 different communities, 3 interviews with HWs (one each from a PFP, PNFP and public facility) and 1 interview with the DHO.

For peer review only

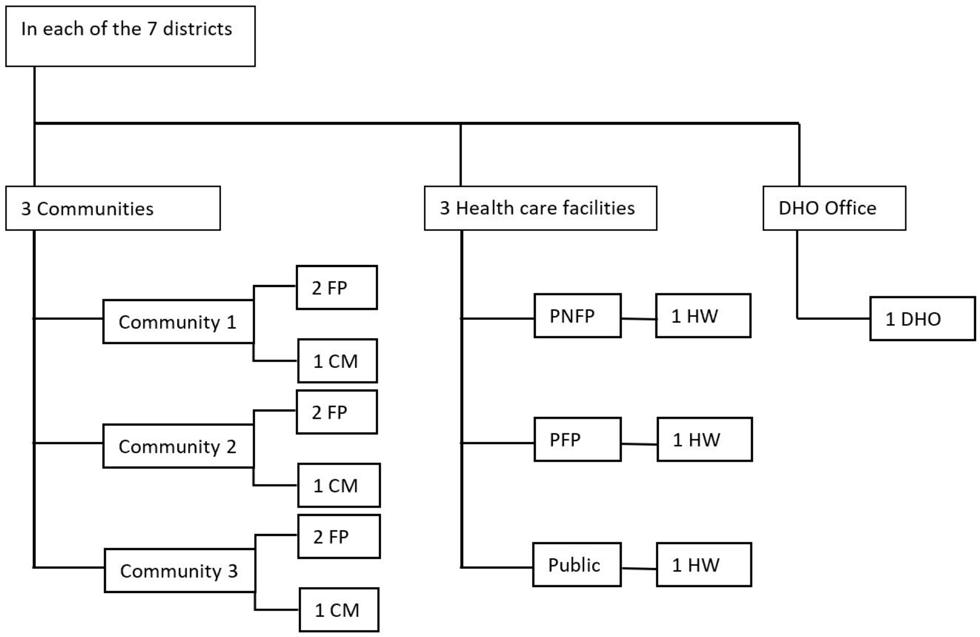

Sampling strategy per district. The goal was to conduct 6 interviews with FPs, 3 interviews with CMs from 3 different communities, 3 interviews with HWs (one each from a PFP, PNFP and public facility) and 1 interview with the DHO.

114x75mm (300 x 300 DPI)

Appendix 1 - Interview Guide District Health Officers

1. In your opinion, what are the main obstacles for the provision of access to healthcare?
2. In your opinion, what are the main obstacles to the provision of surgical care?
3. How do the supply chains for medical equipment work?
4. How do the supply chains for medication work?
5. Which interventions are planned in order to improve access to healthcare, especially to surgical care?
6. Are there campaigns or continuous educational programmes to inform the public about surgical conditions and access to surgical care? Have they proven effective?
7. How much importance is given to the provision of surgery in policy making?
8. What do you see as the most effective measure to increase the surgical workforce?
9. In your opinion, what would be the most effective way for NGOs involved in health care to support the local structures?
10. How could short stays of specialists, e.g. for eye or reconstructive surgery or fistula repair, be integrated into public surgical care for all?

For peer review only

For peer review only - http://bmjopen.bmj.com/site/about/guidelines.xhtml

Appendix 2 - Interview Guide Health professionals

Age:

Gender:

Profession:

Place of work:

Position:

Time period of work at current work place:

1. Please tell me about your personal experience with the provision of surgical care.
2. What are the key obstacles that [profession of interviewee] face at this health care facility?
3. What are the main difficulties in the provision of surgical care?
4. How do you deal with these difficulties?
5. How is the aftercare of surgical procedures organised and do you think it works effectively?
6. According to your experience with surgical care, what do you think are the main areas that need to be improved on?
7. What do you perceive as the main difficulties for patients?
8. In your opinion, what is the best way to address these difficulties for the patients?
9. Would you say patients trust in the expertise of health care professionals? How do patients perceive surgical conditions and treatment in your experience?
10. What are areas that you would describe as well-functioning when it comes to surgical care?

For peer review only

Appendix 3 - Interview Guide – Community Members (Former patients)

Age:

Education:

Occupation:

When were you operated?

Surgical condition:

Where were you operated from?

Length of hospital stay:

1. Please tell me about your experience with surgery.
2. How much time passed from your first symptoms to the diagnosis? And from the diagnosis to the surgical treatment?
3. Were you at any point unsure whether to have surgery and if yes why?
4. Did you consult any party outside of the hospital concerning the health condition you were in?
5. Who took the final decision of whether you would have the surgery?
6. Are you covered by a health insurance?
7. Were there any struggles to reach the hospital?
8. How did the health workers treat you? Were you respected? Were you taken care of?
9. How well did you feel informed about the procedure?
10. How successful was the operation in your opinion?
11. How was the aftercare of the operation? Where was it done? Did you face any struggles taking care of the wound after being discharged from hospital? Did you go through any episodes of extreme pain after the operation?
12. How did your operation change your attitude towards surgery? [This question was amended and changed to: If someone in your family needed to have surgery, what would you advise them?]
13. Throughout the entire process what were the main struggles you faced? In your opinion, which suggestions do you have to improve health facilities in Uganda or your community?
14. In which way did your community support you during that operation and your stay at the hospital?
15. Do you have any questions concerning what we have discussed with you?

For peer review only - http://bmjopen.bmj.com/site/about/guidelines.xhtml

Appendix 4 - Interview Guide – Community Members (general population)

Age:

Education:

Occupation:

1. Please tell me about your thoughts on surgery.
2. What do you think are the biggest barriers to surgical care that members of your community face?
3. Do you know where the closest surgical facility is and how long would it take you to get there?
4. What do you think are health problems for which surgery is needed?
5. Do you go to healthcare facilities regularly for check-up or only in case you experience symptoms?
6. How would you estimate the financial impact of a surgical operation on your livelihood?
7. Are you covered by a health insurance?
8. Would you be concerned if you were told by a healthcare provider that you had to have surgery?
9. If yes, what would be your major concerns?
10. Which things would you take into consideration before opting for surgery?
11. In your opinion, are patients treated right by healthworkers at the hospital?
12. How do people in this community support each other in case someone needs to go to the hospital?
13. Do you think consultations with traditional healers are helpful for possible surgical conditions?
14. Do you have traditional healers in your area? If yes, why do you think patients first consult them instead of going to the health facility directly? For the treatment of which cases are these traditional healers best known for?
15. Which measures would improve access to surgical care for your community in your opinion?

For peer review only

For peer review only - http://bmjopen.bmj.com/site/about/guidelines.xhtml

Standards for Reporting Qualitative Research (SRQR)*

http://www.equator-network.org/reporting-guidelines/srqr/

Title and abstract

	Page/line no(s).
**Title** - Concise description of the nature and topic of the study Identifying the study as qualitative or indicating the approach (e.g., ethnography, grounded theory) or data collection methods (e.g., interview, focus group) is recommended	3/3-5
**Abstract** - Summary of key elements of the study using the abstract format of the intended publication; typically includes background, purpose, methods, results, and conclusions	3/8-32

Introduction

**Problem formulation** - Description and significance of the problem/phenomenon studied; review of relevant theory and empirical work; problem statement	4/5-38
**Purpose or research question** - Purpose of the study and specific objectives or questions	4/34-38

Methods

**Qualitative approach and research paradigm** - Qualitative approach (e.g., ethnography, grounded theory, case study, phenomenology, narrative research) and guiding theory if appropriate; identifying the research paradigm (e.g., postpositivist, constructivist/ interpretivist) is also recommended; rationale**	4/43-49
**Researcher characteristics and reflexivity** - Researchers' characteristics that may influence the research, including personal attributes, qualifications/experience, relationship with participants, assumptions, and/or presuppositions; potential or actual interaction between researchers' characteristics and the research questions, approach, methods, results, and/or transferability	14/42-44
**Context** - Setting/site and salient contextual factors; rationale**	4/55-59
**Sampling strategy** - How and why research participants, documents, or events were selected; criteria for deciding when no further sampling was necessary (e.g., sampling saturation); rationale**	4/60-5/17
**Ethical issues pertaining to human subjects** - Documentation of approval by an appropriate ethics review board and participant consent, or explanation for lack thereof; other confidentiality and data security issues	15/14-19
**Data collection methods** - Types of data collected; details of data collection procedures including (as appropriate) start and stop dates of data collection and analysis, iterative process, triangulation of sources/methods, and modification of procedures in response to evolving study findings; rationale**	5/49-6/4

For peer review only

For peer review only - http://bmjopen.bmj.com/site/about/guidelines.xhtml

**Data collection instruments and technologies** - Description of instruments (e.g., interview guides, questionnaires) and devices (e.g., audio recorders) used for data collection; if/how the instrument(s) changed over the course of the study	5/23-45
**Units of study** - Number and relevant characteristics of participants, documents, or events included in the study; level of participation (could be reported in results)	6/16
**Data processing** - Methods for processing data prior to and during analysis, including transcription, data entry, data management and security, verification of data integrity, data coding, and anonymization/de-identification of excerpts	6/3-4
**Data analysis** - Process by which inferences, themes, etc., were identified and developed, including the researchers involved in data analysis; usually references a specific paradigm or approach; rationale**	6/8-12
**Techniques to enhance trustworthiness** - Techniques to enhance trustworthiness and credibility of data analysis (e.g., member checking, audit trail, triangulation); rationale**	

Results/findings

**Synthesis and interpretation** - Main findings (e.g., interpretations, inferences, and themes); might include development of a theory or model, or integration with prior research or theory	8/27-32
**Links to empirical data** - Evidence (e.g., quotes, field notes, text excerpts, photographs) to substantiate analytic findings	8/34-12/21

Discussion

**Integration with prior work, implications, transferability, and contribution(s) to the field -** Short summary of main findings; explanation of how findings and conclusions connect to, support, elaborate on, or challenge conclusions of earlier scholarship; discussion of scope of application/generalizability; identification of unique contribution(s) to scholarship in a discipline or field	12/24-14/25
**Limitations** - Trustworthiness and limitations of findings	14/27-50

Other

**Conflicts of interest** - Potential sources of influence or perceived influence on study conduct and conclusions; how these were managed	15/28-30
**Funding** - Sources of funding and other support; role of funders in data collection, interpretation, and reporting	15/34

*The authors created the SRQR by searching the literature to identify guidelines, reporting standards, and critical appraisal criteria for qualitative research; reviewing the reference lists of retrieved sources; and contacting experts to gain feedback. The SRQR aims to improve the transparency of all aspects of qualitative research by providing clear standards for reporting qualitative research.

For peer review only

For peer review only - http://bmjopen.bmj.com/site/about/guidelines.xhtml

**The rationale should briefly discuss the justification for choosing that theory, approach, method, or technique rather than other options available, the assumptions and limitations implicit in those choices, and how those choices influence study conclusions and transferability. As appropriate, the rationale for several items might be discussed together.

Reference:

O'Brien BC, Harris IB, Beckman TJ, Reed DA, Cook DA. **Standards for reporting qualitative research: a synthesis of recommendations.** *Academic Medicine*, Vol. 89, No. 9 / Sept 2014 DOI: 10.1097/ACM.0000000000000388

For peer review only

For peer review only - http://bmjopen.bmj.com/site/about/guidelines.xhtml

BMJ Open

Acceptability of surgical care in Uganda: A Qualitative Study on Users and Providers

Journal:	*BMJ Open*
Manuscript ID	bmjopen-2022-070479.R2
Article Type:	Original research
Date Submitted by the Author:	14-Jun-2023
Complete List of Authors:	Rauschendorf, Paula; University of Bonn, Section Global Health, Institute of Hygiene and Public Health<br>Nume, Rosette; Makerere University, School of Women and Gender Studies<br>Bruchhausen, Walter; University of Bonn, Section Global Health, Institute of Hygiene and Public Health
**Primary Subject Heading**:	Global health
Secondary Subject Heading:	Qualitative research, Surgery, Health services research
Keywords:	SURGERY, Organisation of health services < HEALTH SERVICES ADMINISTRATION & MANAGEMENT, Clinical governance < HEALTH SERVICES ADMINISTRATION & MANAGEMENT, Quality in health care < HEALTH SERVICES ADMINISTRATION & MANAGEMENT

SCHOLARONE™
Manuscripts

I, the Submitting Author has the right to grant and does grant on behalf of all authors of the Work (as defined in the below author licence), an exclusive licence and/or a non-exclusive licence for contributions from authors who are: i) UK Crown employees; ii) where BMJ has agreed a CC-BY licence shall apply, and/or iii) in accordance with the terms applicable for US Federal Government officers or employees acting as part of their official duties; on a worldwide, perpetual, irrevocable, royalty-free basis to BMJ Publishing Group Ltd ("BMJ") its licensees and where the relevant Journal is co-owned by BMJ to the co-owners of the Journal, to publish the Work in this journal and any other BMJ products and to exploit all rights, as set out in our licence.

The Submitting Author accepts and understands that any supply made under these terms is made by BMJ to the Submitting Author unless you are acting as an employee on behalf of your employer or a postgraduate student of an affiliated institution which is paying any applicable article publishing charge ("APC") for Open Access articles. Where the Submitting Author wishes to make the Work available on an Open Access basis (and intends to pay the relevant APC), the terms of reuse of such Open Access shall be governed by a Creative Commons licence – details of these licences and which Creative Commons licence will apply to this Work are set out in our licence referred to above.

Other than as permitted in any relevant BMJ Author's Self Archiving Policies, I confirm this Work has not been accepted for publication elsewhere, is not being considered for publication elsewhere and does not duplicate material already published. I confirm all authors consent to publication of this Work and authorise the granting of this licence.

For peer review only

Acceptability of surgical care in Uganda: A Qualitative Study on Users and Providers

1. Corresponding author
   Paula Kristina Rauschendorf
   Section Global Health
   Institute of Hygiene and Public Health
   University of Bonn
   Bonn, Germany
   p.rauschendorf@outlook.de
   Tel. +1(617)201-1231

2. Rosette Nume
   School of Women and Gender Studies
   Makerere University
   Kampala, Uganda

3. Prof. Dr. med. Walter Bruchhausen Dipl.-Theol. M.Phil.(Glasg.)
   Section Global Health
   Institute of Hygiene and Public Health
   University of Bonn
   Bonn, Germany

Keywords: Health education and promotion, Surgery, Qualitative Study, Health services research

Word count: 5443

For peer review only

Acceptability of surgical care in Uganda: A qualitative study on users and providers

Abstract

Objectives This study was conducted to assess acceptability of surgical care in Eastern Uganda and enable better allocation of resources and to guide health policy towards increased surgical care seeking.

Design This qualitative study utilized semi-structured in-depth interviews that were transcribed and analysed by coding according to grounded theory.

Setting The study was set in Eastern Uganda in the districts of Jinja, Mayuge, Kamuli, Iganga, Luuka, Buikwe and Buvuma.

Participants Interviews were conducted with 32 past surgical patients, 16 community members who had not undergone surgery, 17 healthcare professionals involved in surgical treatment, and 7 District Health Officers or their deputies.

Results The five intersecting categories that emerged were health literacy, perceptions, risks and fears, search for alternatives, care/treatment and trust in healthcare workers. It was also demonstrated that considering the user and provider side at the same time is very useful for a more extensive understanding of surgical care seeking behaviour and the impact of user-provider interactions or lack thereof.

Conclusion While affordability and accessibility are well defined and therefore easier to assess, acceptability is a much less quantifiable concept. This study breaks it down into tangible concepts in the form of five categories, which provide guidance to future interventions regarding surgical care acceptability. We also demonstrated that multiple perspectives are beneficial to understanding the multifactorial nature of health care seeking and provision.

Strengths and limitations

- This study provides multiple perspectives by interviewing local health officials, (potential) users and providers of surgical care.
- Among the interviewees of each group, diversity was created through purposive sampling.
- This study is limited by its small sample size and geographical area, despite its attempt for representativity.
- Some questions needed re-phrasing after analysis of the first set of interviews following grounded theory due to them being misunderstood

For peer review only

Introduction

In 2015, the Lancet Commission Report on Global surgery highlighted the inequity of the global burden of surgical disease and access to care [1]. Surgical care, which is defined as “any intervention directed at reducing the disability or premature death associated with a surgical condition” [2], has been found to be highly effective in reducing the burden of disease and disability-adjusted life years lost [3]. Uganda faces a high unmet need for surgery [4–6], with few operating theatres (0.2 major OTs per 100,000 people) [7] and a low surgeon density of 0.73 surgeons per 100,000 persons [6]. Wards are overbooked [8] and shortages of necessities such as water, electricity, equipment, and limited access to blood banks further hinder safe surgical care [9].

The inequity in burden of surgical disease and access to care, in the context of Universal Health Coverage (UHC) as a target of the Sustainable Development Goal 3 [10] underscores the importance of *accessible*, *affordable* and *acceptable* surgical care. While accessibility [11–13] and affordability [14] of surgical care have been thoroughly researched in Uganda, acceptability of surgical care has not been a focus.

Acceptability of care was defined by Sekhon et al. as "a multi-faceted construct that reflects the extent to which people delivering or receiving a healthcare intervention consider it to be appropriate, based on anticipated or experienced cognitive and emotional responses to the intervention.” [15] It is influenced by a multitude of factors such as the quality of healthcare facilities, availability and attitudes of and towards healthcare personnel, perceptions, risks and fears associated with healthcare, available alternatives and health literacy, which can be defined as “the degree to which individuals have the ability to find, understand, and use information and services to inform health-related decisions and actions for themselves and others.” [16]

Research on acceptability of care can provide insights into reasons for low uptake of surgical care and inform resource allocation and health policies. Acceptability of care needs to be assessed from multiple perspectives: patients, as well as the broader public, health care providers and health officials can each provide a unique viewpoint. Therefore, this study analyzes interviews with former surgical patients, community members, surgical healthcare professionals, and District Health Officers.

Methods

Study design

This qualitative study utilized semi-structured in-depth interviews that were transcribed, if needed translated and analysed by coding according to grounded theory. Grounded theory is a qualitative research method aimed at developing theories from data and their analysis [17–19] that can increase understanding of social behaviours and processes, e.g. in patient care [20]. Due to its inductive nature, it is often used in fields with little prior research.

Patient and public involvement

There was no patient or public involvement in the study prior to data collection.

Setting and participants

The interviews were conducted from August to September 2019 in the districts of Jinja, Mayuge, Kamuli, Iganga, Luuka, Buikwe and Buvuma. They are located in South-Eastern Uganda and have an estimated population of 2.84 million [21]. The Eastern region of Uganda has a significantly higher poverty rate than the national average. [22]

We interviewed members of four groups in each district:

For peer review only

- six former surgical patients (FP) and
- three community members who had not had surgery (CM) from three different communities, which were selected purposively with regards to distance to the next surgery providing health care facility and distance from the main road. One community was chosen near a surgical facility, one was chosen to be further from a facility but along the main road and one was chosen further away from both the facility and the main road.
- three healthcare workers involved in surgical care (HW), including representatives from a private for profit (PFP), private non-for-profit (PNFP) and government (public) facility. In districts where PFP or PNFP facilities were lacking, either two interviews were conducted at a government facility, or one interview was conducted in the district. To ensure diversity and minimize selection bias, different medical cadres were interviewed in each sector, following purposive sampling.
- the District Health Officers (DHO).

Figure 1

Interview guide

The process of developing the interview guides involved reviewing relevant literature, identifying key concepts or themes, and crafting open-ended questions to elicit in-depth responses from the interviewees. The final interview guides were structured in a logical sequence to facilitate a smooth flow of the interviews and to ensure that all relevant topics were covered. The interview guides served as a flexible tool that allowed for probing and follow-up questions based on the responses of the interviewees, ensuring that a rich and comprehensive data set was collected during the interviews. After analysis of the first set of interviews, some questions were rephrased for clarity.

During the interviews, FPs were asked about their experience with surgery, delays in healthcare seeking, reasons for uncertainty, consultations outside of biomedical healthcare, decision making, the process of their surgical treatment, changes in surgery perception, community support and suggestions for improvement of health facilities. CMs were asked about their thoughts on surgery and their perceived struggles, health behaviours, knowledge on surgical conditions, financial impact, health insurance coverage and their concerns pertaining to surgery.

HWs were asked about their experience in providing surgical care, difficulties, improvements, and patient perceptions. Interviews with DHOs commenced with a question about obstacles in providing access to healthcare, particularly surgical care, and followed by questions about supply chains, health education campaigns, the surgical workforce and ways of involvement of NGOs.

For full interview guides please refer to Appendix 1-4.

Data collection

Overall, 72 interviews were conducted. Community interviews were conducted in Lusoga. Interviews with HWs and DHOs were conducted in English or Lusoga depending on preference.

The DHO was approached for an interview and simultaneously permission was obtained to interview HWs in the district. Then HWs were invited to participate in the study at their respective healthcare facilities. Community members were engaged and asked if they had undergone surgery or could refer to someone who had. This process was repeated until two FPs and one CM were recruited.

Theoretical saturation was reached towards the end of sampling, defined as the “point in category development at which no new properties, dimensions, or relationships emerge during analysis” [23] and “new data collection (indicated by theoretical sampling) does not lead to any further changes to

For peer review only

the theory" [24]. This suggests that the sample size was sufficient for addressing the research questions and achieving the research objectives.

Interviews were audio recorded, transcribed verbatim and anonymized. Translations to English were performed if the original language of the interview was not English.

Data analysis

Data was analysed using MAXQDA Pro Analytics. MAXQDA is a qualitative data analysis software that provides various tools and features for analysing qualitative data. The software allows researchers to organize, code, and analyse text, audio, video, and image data in a systematic and rigorous manner. Interview transcripts were coded according to grounded theory by finding the key words in each section of the interviews. Codes were grouped into sub-categories and sorted into larger categories.

Results

Interviews were conducted with 33 FP, 16 CM, 17 HW and 7 DHO or their deputies.

Demographic data of the sample can be found in Table 1 and 2.

For peer review only

Table 1. Demographic characteristics of FP and CM

	Former Patients		Community members	
**Characteristic**		**Percentage**		**Percentage**
**Sex**				
Female	21	63.6	10	62.5
Male	12	36.4	6	37.5
**Age (years)**				
<20	3	9.1	0	0.0
21-40	12	36.4	3	18.8
41-60	12	36.4	10	62.5
61-80	5	15.2	3	18.8
>80	1	3.0	0	0.0
**Level of education**				
Tertiary	3	9.1	2	12.5
Secondary	8	24.2	3	18.8
Primary	9	27.3	11	68.8
None	5	15.2	0	0.0
No answer	8	24.2	0	0.0
**Employment status**				
Self-employed	2	6.1	4	25.0
Unemployed	16	48.5	11	68.8
Formal employment	6	18.2	0	0.0
Retired	1	3.0	0	0.0
No answer	8	24.2	1	6.3
**Time of surgery**				
2015-2019	15	45.5		
2010-2014	4	12.1		
2005-2009	3	9.1		
Before 2005	1	3.0		
No answer	10	30.3		
**Facility type**				
Public	16	48.5		
PNFP	5	15.2		
PFP	8	24.2		
No answer	4	12.1		

For peer review only - http://bmjopen.bmj.com/site/about/guidelines.xhtml

Table 2. Demographic characteristics of HW

	Healthcare workers	
**Characteristic**		**Percentage**
**Sex**		
Female	4	23.5
Male	13	76.5
**Profession**		
Nurse	1	5.9
Midwife	1	5.9
Theatre assistant	1	5.9
Medical officer	4	23.5
Medical doctor	6	35.3
Surgeon	3	17.6
Obstetrician	1	5.9
**Facility type**		
Public	8	47.1
PNFP	5	29.4
PFP	4	23.5

During the analysis, five dimensions of acceptability of surgical care emerged. *Health literacy* was found to impact healthcare seeking behaviour and shape expectations about surgical care, which were further influenced by *fears and perceptions of surgery*. Comparisons were made between surgical care and *alternative healthcare options*. Trust in the *healthcare worker* as well as *care and treatment* emerged as important categories influencing acceptability.

Health literacy: impact and improvement

Low levels of health literacy impacted healthcare seeking negatively according to many participants from all groups. A DHO lamented that patients did not know the "*timely need for coming for healthcare*". HWs supported this and explained that "*people don't know that they require surgery*" (surgeon, PNFP) or "*the impact of their condition*" (medical doctor (MD), PNFP). An FP mentioned that a mass in his spleen was discovered during an intestinal obstruction surgery one year before, but he did not seek further medical evaluation afterward.

Many FPs reported waiting for a long time before seeking care, as one said: "*I delayed a bit. I think it was around [...] 10 years*" (FP, PFP). Along with other participants, another FP attributed the delay to his "*ignorance, because I wasn't aware the illness needed a medical treatment*" (FP). Some participants described self-medicating, as in this example: "*When I am sick, I just buy medicine of 2000 to 3000 shillings and if that sickness doesn't respond to the treatment, I continue changing the type of treatment until I get better.*" (CM)

Low levels of general education were discussed, since "*perceptions are about education*" (medical officer (MO), public). A HW described struggling to find a comprehensible way to explain surgical conditions to the patients. CMs similarly said: "*after taking blood samples from us, there's nothing you can understand*" (CM) and "*those who never went to school don't know anything and you have to believe all what the healthcare workers tell you*" (CM).

While many CMs demonstrated knowledge on obstetric conditions and hernia, other conditions such as goitre, ulcer, and cancer were less frequently mentioned. A HW attributed this to the fact that

For peer review only

"*surgery doesn't have enough advocacy, but obstetric, emergency obstetric care has received a lot of advocacy and a lot of funding*" (MO, public). He described the increase in health education campaigns around obstetric emergencies had led to women "*coming to the facilities, seeking care, surgical care compared to general surgery*." (MO, public). Another MO (PNFP) confirmed the importance of such campaigns for recruiting patients.

Therefore, more health education specific to surgery was proposed to improve surgical care seeking. Suggestions included campaigns broadcasted via radio and televisions as well as outreaches and village health teams (VHTs) "*to identify some of these complications – the illnesses – and educate, give some health education and refer these people to where they can get services*" (Surgeon, PFP). One HW raised concerns that educating the population had to be linked with building surgical capacity to accommodate the anticipated increase in surgical patients.

Perceptions and Fears of Surgery

Surgery was perceived by CMs and FPs as a way of "*reducing the sickness that is bothering you*" (FP, PNFP) by "*cutting you open to see the cause of sickness and remove the unwanted stuff*" (FP, PFP), oftentimes with urgency: "*the patient is in a critical and not understandable condition, then why can that patient not be taken to be operated*" (CM). Surgical care was described as a last resort by CMs and FPs, often only an option "*when the condition that calls for the operation is very extremely severe, when the pain is so so so beyond, without giving you time to eat, drink or walk, I mean when the illness is at its maximum, then there you can decide to go for the operation*" (CM).

The risk associated with surgery was often named as "*you can come back or not come back*" (CM). A surgeon (PNFP) attributed these sceptical attitudes to the fact that "*in the past the results from surgery have not been good. It has been between life and death*." One of the DHOs mentioned that cases with complications are overly emphasised causing a prevalent misconception that surgery is unsafe. Seeming medical malpractice in anaesthesia was described by one FP (PFP): "*When I was still half sedated, they cut me but the way they pulled my scrotum with much force I felt terrible pain in the intestines*".

Participants mentioned the fear that surgeons would leave instruments or swabs in the patient with possible complications and re-operations. One recounted that the people "*normally say that when they go for surgery, they make you more disabled not allowed to do certain work*" (CM). Another example was the fear that putting a patient "*on oxygen means he's going to die*" (nurse, PNFP). Also, there were "*rumours that healthcare workers steal certain organs from the patients*" (CM) which led to fear among the community. One FP (public) suggested having a relative of the patient in the theatre to "*see the procedures of the operation and what they have removed from the patient*".

One midwife (PFP) saw an increase in trust after successful operations, which was also apparent in some interviews with FPs: "*healthcare providers can operate and after the operation you can wake up and resume with normal walking, healthy person, and the illness is taken away you can become better, and you live a healthy life.*" (FP, PNFP) Another FP (PFP) described how she was asked after her surgery "*not to scare my fellow women that the health facility was a terrible place; they told me to advise my friends to go for medical procedures*". This demonstrates that staff feels the need for improving the reputation of surgical treatment. As a way of counteracting patient fear, effective pre-operative counselling was suggested by FPs and healthcare providers.

Understanding of Illness and the Search for Alternatives

Surgical conditions as seen from a biomedical perspective were mostly regarded by FPs and CMs as necessitating biomedical treatment, and many participants named biomedical facilities as their only source of healthcare. Patients mostly became aware that their condition was surgical after it had

For peer review only

been identified as such by a healthcare worker. One FP (PNFP) stated: "*sickness that is meant for operation, still goes for operation, there is no alternative*". In contrast to the view that surgical conditions always necessitate surgery, one medical officer described that pertaining to surgical diagnosis "*they [the patients] think there is something that you have not done. They think you should have given some medication for that condition*."

Herbal medicine, for example was described as a way of preventing surgery under certain circumstances: "*I was to have a normal delivery if I used the local herbs*" (FP, public). It was only mentioned in the context of chronic conditions like hernia, not as an alternative in emergency or trauma cases. An FP described "*someone went to those healers and was given herbs and his hernia disappeared, so I also went there and used those herbs for one day but when I noticed no improvement, I gave up on them*." (FP, PFP). Another example was given by an FP (PFP) with haemorrhoids, who was later operated: "*when I applied the herbs, the piles went back but after some time, it re-occurred, and when I asked them about that, they told me that, the piles can only be healed with traditional medicine and so I continued with that medicine.*" HWs expressed frustration but blamed it on the "*traditional healers or herbalists who keep misguiding or giving false information to our clients*" (surgeon, PFP) and the lack of knowledge concerning surgical conditions.

Similarly, when asked about traditional healers in the area, many respondents voiced distrust because they would not "*reveal that your condition needs surgical treatment. When they want money, they just divert you from seeking medical care, yet they can't treat the problem at hand.*" (CM) However, some participants also saw fault with the patients that sometimes "*cannot differentiate a medical problem from a traditional one*" (CM) and "*think that they have been bewitched, even if the sickness is not traditional*" (FP). This shows an understanding of ill-health and healing of two separate spheres with different illnesses and their corresponding healthcare, i.e. an alternative between "medical" and "traditional".

Differing from this understanding of two completely distinct types of illness and treatment, a CM argued "*those other things of bewitching can come later after going to the health facility*" (CM). Here the common view of complementary functions is expressed that biomedicine may – even quickly – remove the damage like hernia, but not the underlying cause like bewitchment, as one said: "*Can a healthcare worker tell you issues of the home? […] But it is the traditional healer to tell you the facts. The healthcare worker can suspect the cause of illness and tell the patient that maybe the patient delayed getting treatment, but when you go to these ones, they can tell you.*" (CM).

Care and Treatment

The received care was commonly described as good. Good was, however, defined differently by FPs, e.g. as not being mistreated or being spoken to "*in a friendly manner*" (FP, PNFP). Others voiced more specific expectations: "*to be cared for, is when the healthcare worker comes in the ward and greets you asking about how the night was, how you feel, the condition of the baby and other issues like that.*" (FP, public) For others the focus was more on the medical aspects like receiving "*timely treatment*" (FP, PFP).

The type of care received was commonly attributed to the patient's financial situation, where paying the asked amount was linked to good care and medical treatment, whereas patients with financial constraints were often mentioned to receive worse treatment. Other FPs attributed the good care they received to personal relationships with the healthcare workers. One FP (PNFP) reported that "*they respected me because first of all I am educated, and I interacted with them in English during all the chats*".

For peer review only

Despite general satisfaction, some complaints were voiced like going through episodes of extreme pain after surgery, some to the extent, that it would prevent future surgical care seeking. Other FPs mentioned mistreatment by healthcare workers, for example, a female FP (public) was on a trolley to the operation theatre for a C-section when she "*felt something like a baby coming out then I widened my legs as if I was going to deliver, then the nurse slapped me for widening the legs, and exposing my nakedness to the people*". The nurse was held accountable for her actions. A CM lamented: "*if you are lucky enough and your patient is operated, they carelessly stich and just dump him/her there for you. They are always not concerned with the aftereffects of the operation, it's you to care for the patient.*"

This alludes to the important role of informal caregivers in Ugandan healthcare. "*They carry the burden, the financial, emotional, and so on*" (surgeon, public). Their importance was highlighted multiple times throughout interviews, for example when one FP (public) said, "*if you have no effective caregiver, it's a big challenge*". The informal caregiver has different responsibilities, mainly taking care of the basic needs of patients, while at the hospital and to provide emotional support. One FP (public) stated, that when patients did not have their treatment readily prepared by their informal caregiver nurses "*just pass to another patient*".

The "*lack of proper trained surgical nursing care*" (medical superintendent) led to some surgeons feeling "*let down by the postoperative care*" (MO, public). A nurse (PNFP) explained: "*we are like 2 or 3 on duty and patients are many and we have to attend to them, sometimes you find that you have not reached up to that peak of caring for the patient*". Therefore, sometimes "*responsibilities channel to the relatives*" (MO, public) that should be carried out by medical personnel. A surgeon noted that this led to problems in the aftercare of operations. HWs also noted that the lack of separate surgical wards was a challenge to providing specialised care. To counteract these challenges, more training, a separate surgical ward and an increase in staff were suggested.

Follow-up of operations was another theme that emerged. Some FPs said they had not been asked back for follow-up. HWs commonly said that review dates were scheduled with patients, but attendance varied. This was a challenge for surgical staff, as doctors were then "*not able to monitor them for any complications*" (Surgeon, PFP). Varying commitment from HWs was apparent, with some recording patients' numbers and calling to check up and some visiting their patients at home. A surgeon (PFP) suggested that regular "*field visits*" could improve follow-up.

Healthcare workers: Trust and Misbehaviour

Closely linked to care and treatment was the patient-healthcare worker relationship. As one CM stated: "*the healthcare worker is the overall in this thing, he/she is the key to everything*". Trust and lack thereof were commonly mentioned.

Most interviewed HWs agreed that they felt trusted by patients. Some FPs described a trustful relationship, with one mentioning being taken care of by a doctor off-duty and another calling the doctor a "*saviour*" (FP, public). One medical officer (public) differentiated: "*Those who come, trust us. What they don't trust is the system. What they don't trust is their government*". Contrastingly, distrust in the surgical skills of healthcare workers was also mentioned with one FP saying: "*most of the healthcare workers are young boys, we don't trust them*". This is caused by the regulation that demands service in remote communities in the first years after graduation. One doctor (PFP) highlighted that this was a major difference to traditional healers, as they "*tend to give more time to their patients actually and convince them adequately*". The overwhelming workload and little time spent with the patient were given as reasons for the lack of trust in the healthcare system and HWs.

HWs were expected to "*first think about people's health, then think about money*" (CM) because "*there are rewards in heaven*" (CM). Work ethics of HWs were a concern to participants from the community, who described nurses and doctors commonly asked for informal payments. One FP (public) described being asked "*to pay her 60,000 shillings for quick treatment and we go home instead of being admitted in the ward*"). Some mentioned, HWs would let the patient die if they could not pay: "*There was that first one I saw, he had even refused to operate me, and if the second one never showed up, I was going to die*" (FP, public). Participants suggested to raise salaries of HWs, so they would stop demanding informal payments.

Medical personnel generally described their own work ethics as high, with one medical officer calling it a "*commitment to serve*". One doctor (PNFP) described having to pay for medical equipment when the patient cannot afford it: "*sometimes it is necessary, where you see that life is more important*". Nevertheless, many saw issues with motivation and work morale in the health workforce in general, attributing it to frustration due to poor salaries, long working hours, poorly equipped facilities, and little opportunity for continued education and mentorship. Scholarships for further education and mentorship were mentioned as important. Training programmes through foreign exchange or as part of surgical camps were seen as beneficial for both sides: "*Probably you might come with the skills where you come from, but the context differs. Then you also have something to learn with us, from us working with limited resources*." (DHO)

Discussion

In providing surgical care to communities, like in Uganda, factors beyond accessibility and affordability necessitate careful consideration. While some commonly used models like the Behavioural Model of Health Service Use by Andersen [25] consider mostly the user side, this study aims to showcase the user, the provider and the health officials' perspective. The five areas found to impact acceptability of surgical care were: health literacy, perceptions of surgery, the search for alternatives, care and treatment and the relationship to the healthcare worker. These categories, however, had some overlap.

Higher health literacy has been shown to increase acceptance of surgical care [26] while limited health literacy has been linked to worse outcomes and lower satisfaction [27] and to overall non-adherence to pre- and postoperative instructions [28]. Participants of our study, especially on the provider side, also viewed health literacy on surgical conditions impactful for improving surgical care seeking. This should be a focus of future interventions, as suggested by Butler et al. in a study where lack of perceived need left 25% of children with surgical needs untreated [29]. Health literacy assessments have been shown to be feasible and to enable increased patient satisfaction [30]. General education and literacy were also discussed by participants as enabling patients to make better decisions on healthcare seeking for surgical conditions, which was also found by Fuller et al. [31] and Davé et. al [32]. Both studies were based on the Surgeons Over Seas Assessment of Surgical Need, a cluster randomized, cross-sectional, national survey employing quantitative methods to assess the prevalence of sugical conditions and related healthcare seeking behaviour.

A thorough understanding of the perceptions of surgery and concepts of illness is necessary to provide acceptable care. The question of what is at stake for those people most concerned by a surgical condition needs to be addressed for meeting important expectations and creating trust. Understandably sceptical attitudes due to former negative experiences with formal education and biomedical healthcare [33] as well as unfavourable surgical outcomes [34] have a large impact on surgical healthcare seeking.

For peer review only

While the surgical treatment was often described as successful by patients, negative perceptions were commonly mentioned. Risks and fears related to surgical interventions act as a barrier for their uptake. Perceptions were also shaped by rumours, like concerns of organ stealing. Rumours like this have existed in many African societies and have been discussed extensively by White [35] and Scheper-Hughes [36]. Other rumours noted were specific to surgical care and to proceedings of an operating room. Improving health literacy, setting a focus on communication with patients and improved education prior to surgery could play an important part in counteracting rumours. It could also reduce self-medication and change the current role of surgery as a last resort or for emergencies only.

With 4.0% of rural children with surgical conditions not receiving care due to fear [37], trust and positive interactions with surgical care providers are important. While most healthcare personnel felt trusted by patients and many patients voiced trust, some scepticism towards surgical skills particularly of medical officers was expressed. Similar concerns about seniority and skill level were voiced by participants in a study by Mwaka et al suggesting that only senior and skilled doctors were regarded as fit to successfully operate cancer patients [38]. Due to lack of specialists, non-specialised personnel commonly conducts surgery in Uganda [39].

Participants in our study, especially medical officers, wished for more mentorship and further specialisation. This would also provide an opportunity for more training on interpersonal skills to improve communication with patients, as suggested by Bohren et. al [40]. Surgical camps were suggested as possible training opportunities. Modern technology was also proposed to improve mentorship, where medical officers in remote areas would be able to consult with colleagues in Uganda or abroad via telecommunication.

Most FPs and CMs recognized the need for operative care in case of surgical conditions. These findings align with results from a previous study investigating surgery beliefs of lay people, where surgery was seen as “the appropriate healing method”, although there was also “fear of negative outcome of surgery” [38]. In our study, some participants mentioned attempting alternative treatments under the category of “traditional medicine”, which was also a common theme among providers. Participants from communities often agreed there were indications for traditional medicine, however rarely including surgical conditions like hernia.

Many participants from all groups highlighted the dangers of misdiagnosis and delay of treatment, which has been previously reported [5, 29] and was discussed by WHO in their traditional medicine strategy [41]. However, patients continue to seek care from traditional healers, which healthcare providers attributed to the fact that traditional healers often spend more time with patients. This could be due to the ratio of 1 traditional healer per population of 500, compared to 1 medical doctor per 25000 people in Uganda [42]. Participants also discussed their role as holistic healers. Some of these features could possibly be a starting point for developing a healthcare system encompassing the underlying needs of patients answered by traditional medicine.

Overall, patients in this study were satisfied with care, but noted that factors such as financial situation, education level and relationships with healthcare workers influenced the type of care received. Similar findings were reported in other studies, citing bribes, socio-economic class, political affiliation, and tribal favouritism [43] as well as social capital and financial leveraging [44] as factors impacting care. Participants in our study linked these behaviours to the work ethics of HWs. While HWs expressed high motivation, many described low morale among the health workforce, due to factors such as low salaries, difficult work environments and lack of mentorship.

Participants described negative interactions between healthcare providers and patients, including harsh treatment and neglect. This is consistent with previous findings from Benin [34]. Kagoya et al.

found a lack of negotiating power among patients at the Ugandan National Referral Hospital caused by “illiteracy, language barriers, low socio-economic status, feeling inferior to HWs and perceived favour to access free services” [43].

Some FPs described situations which could be interpreted as malpractice or errors in surgical care. A study on general medical errors found how they occurred and that healthcare professionals were mostly aware of them [45]. In regards to surgical errors, “inappropriate pre-operative management, omitting preoperative investigations and omitting post-operative notes“ [46] were most commonly found by reviewing patient records. A lack of error reporting on medication errors was also found by Kiguba et. al. [47].

Post-surgical care and follow-up were seen as major challenges by patients and healthcare providers. Lack of trained nursing staff leads to task shifting to informal caregivers, as highlighted by participants. In post-operative caregiving, informal caregivers often lack the necessary training, education and understanding of medical conditions necessary to provide the best care possible. Formerly proposed caregiver training to increase capacity of post-surgical care [48] was not suggested by participants in this study, who laid more focus on increasing the surgical workforce.

Previous research suggests that receiving follow-up care is strongly dependent on socio-economic status and distance to access points [48]. Our findings support this. Healthcare professionals in our study suggested taking phone numbers of patients and making fixed appointments to combat this problem. Home visits were mentioned as ideal, but not feasible. A study on maternal and new-born care practices also suggests that a combination of home visits by VHTs and mobile phone consultations improve care [49]. Interventions linked to VHTs should be strengthened and possibly expanded to include regular basic health check-ups to facilitate earlier interventions on complications and remissions.

Limitations

In its aim to provide a broad overview of perspectives on surgical care, this study is limited by its small sample size and geographical area, despite its attempt for representativity. However, the broader categories that emerged may be applicable to the country and could even find application beyond. The study also showed that grounded theory can be applied to study acceptability of care in specific contexts.

In the first interviews participants were not asked to provide the year of their surgery. In the selection of participants, no exclusion criteria were defined regarding the year of surgery. While this approach may have limited representativity for current surgical care, it was intentional as it was believed that experiences from the past may still shape perceptions and attitudes within the communities, even if the surgery occurred long ago.

The educational, social science, medical, and cultural backgrounds of the researchers conducting the interviews, being a Ugandan graduate student and a German medical doctoral student, may have created a sense of authority or power dynamic that could have impacted the participants' answers. This introduces the potential for observer bias in the study, as the presence of the researchers during the interviews could have influenced the responses of the participants.

Concerning both, reliability and validity, some questions needed re-phrasing after grounded theory analysis of the first set of interviews since they had been misunderstood.

For peer review only

Conclusion

This study emphasises the need to consider the views of users, providers and local health officials for a comprehensive understanding of factors influencing the acceptability of surgical care. Expectations of surgical care emerged to be shaped by *health literacy* and *perceptions* of illness and surgery. Extensive information and health education campaigns on surgical conditions could improve healthcare seeking and empowerment of patients and their relatives. Reasons for seeking out *alternative treatments* can provide insights for future interventions. The *care and treatment* at large as well as the *relationship with the healthcare worker* impacted acceptability. Negative experiences by patients could be addressed by improving the training of medical personnel, enhancing interpersonal skills and promoting a respectful environment in hospitals and healthcare settings. The needs of providers should be taken into consideration to improve motivation and workplace satisfaction. Structural improvements and an increase in qualified staff are the basis for a work environment in which acceptability can be given the necessary attention. Finally, our findings suggest the need for more extensive research on the underlying mechanisms of acceptability of surgical care, as an important foundation to tackle delays in surgical care seeking.

Ethical considerations

Permission to conduct the interviews was obtained from the Ugandan National Council for Science and Technology after receiving ethical clearance from Mildmay Uganda Research Ethics Committee under #REC REF 0102-2019. For interviews with DHOs permission was given by the Ministry of Health. Furthermore, we asked permission of DHOs to conduct interviews with HW in their district. All participants provided informed consent to be eligible to participate in this study.

Data Sharing statement

We provided all relevant excerpts within the manuscript as raw data cannot be shared publicly due to the significant and valid confidentiality concerns of interviewees, small sample size, and ethics approval requirements.

Conflict of Interest

The author(s) certify that they have no conflict of interest in the subject matter or materials discussed in this manuscript.

Funding

The study was funded by the Section Global Health at the University Hospital Bonn.

Author Contributions

PR, a German medical doctoral student at the time of the study, and WB, a professor for Global Health at the Universities of Cologne and later Bonn, developed the study design and the interview guides. Data collection was carried out collectively by PR and RN, a master's student at the School of Women and Gender Studies. PR then conducted the data analysis and prepared the manuscript. WB oversaw all aspects of this research. All authors contributed to the article and approved the submitted version.

Acknowledgements

We thank Global Health at University Hospital Bonn for the financial support, Ando-Modular aid e.V. for the inspiration for this study, Hatiika Namyalo for helping us navigate the Ugandan health care system and Grace Meara for helpful discussions.

For peer review only

REFERENCES

1 Meara JG, Leather AJM, Hagander L, et al. Global Surgery 2030: evidence and solutions for achieving health, welfare, and economic development. *Lancet* 2015;386(9993):569–624. http://www.sciencedirect.com/science/article/pii/S014067361560160X.

2 Bickler S, Ozgediz D, Gosselin R, et al. Key concepts for estimating the burden of surgical conditions and the unmet need for surgical care. *World J Surg* 2010;34(3):374–80.

3 WHO Executive Board. Strengthening emergency and essential surgical care and anaesthesia as a component of universal health coverage: Report by the Secretariat 2014.

4 Butler EK, Tran TM, Nagarajan N, et al. Epidemiology of pediatric surgical needs in low-income countries. *PloS one* 2017;12(3):1-9. doi:10.1371/journal.pone.0170968 [published Online First: 4 March 2017].

5 Tran TM, Fuller AT, Butler EK, et al. Surgical need among the ageing population of Uganda. *Afr Health Sci* 2019;19(1):1778–88.

6 Farber SH, Vissoci JRN, Tran TM, et al. Geospatial Analysis of Unmet Surgical Need in Uganda: An Analysis of SOSAS Survey Data. *World J Surg* 2017;41(2):353–63.

7 Linden AF, Sekidde FS, Galukande M, et al. Challenges of surgery in developing countries: a survey of surgical and anesthesia capacity in Uganda's public hospitals. *World journal of surgery* 2012;36(5):1056–65.

8 Nwanna-Nzewunwa OC, Ajiko MM, Kirya F, et al. Barriers and facilitators of surgical care in rural Uganda: a mixed methods study. *The Journal of surgical research* 2016;204(1):242–50. doi:10.1016/j.jss.2016.04.051 [published Online First: 28 July 2016].

9 Albutt K, Yorlets RR, Punchak M, et al. You pray to your God: A qualitative analysis of challenges in the provision of safe, timely, and affordable surgical care in Uganda. *PloS one* 2018;13(4):1-16. doi:10.1371/journal.pone.0195986 [published Online First: 18 April 2018].

10 UN General Assembly. Transforming our World: The 2030 Agenda for Sustainable Development 2015.

11 Albutt K, Punchak M, Kayima P, et al. Access to Safe, Timely, and Affordable Surgical Care in Uganda: A Stratified Randomized Evaluation of Nationwide Public Sector Surgical Capacity and Core Surgical Indicators. *World J Surg* 2018;42(8):2303–13.

12 Albutt K, Drevin G, Yorlets RR, et al. 'We are all serving the same Ugandans': A nationwide mixed-methods evaluation of private sector surgical capacity in Uganda. *PLoS One* 2019;14(10):e0224215.

13 Albutt K, Namanya D, Anderson G, Kayima P. Uganda. In: Johnson W, Lin Y, Mukhopadhyay S, Meara J, ed. Surgical care systems strengthening: developing national surgical, obstetric and anaesthesia plans. Geneva: World Health Organization 2017:4–7.

14 Anderson GA, Ilcisin L, Kayima P, et al. Out-of-pocket payment for surgery in Uganda: The rate of impoverishing and catastrophic expenditure at a government hospital. *PloS one* 2017;12(10):1-13.

15 Sekhon M, Cartwright M, Francis JJ. Acceptability of healthcare interventions: an overview of reviews and development of a theoretical framework. *BMC Health Serv Res* 2017;17(1):88. doi:10.1186/s12913-017-2031-8 [published Online First: 26 January 2017].

16 U.S. Department of Health and Human Services. Health Literacy in Healthy People 2030 - Healthy People 2030 | health.gov 2023. Available at: https://health.gov/healthypeople/priority-areas/health-literacy-healthy-people-2030 Accessed April 22, 2023.

17 K Charmaz. Constructing grounded theory: Charmaz K. Constructing grounded theory. London: Sage publications; 2014 2014.

For peer review only

18 J Corbin. Basics of qualitative research: Techniques and procedures for developing grounded theory: Corbin J, Strauss A. Basics of qualitative research: Techniques and procedures for developing grounded theory. Thousand Oaks: Sage publications; 2014 2014.

19 BG Glaser. The discovery of grounded theory: Strategies for qualitative research: Glaser BG, Strauss AL. The discovery of grounded theory: Strategies for qualitative research. USA: Transaction Publishers; 2009 2009.

20 Higginbottom G, Lauridsen EI. The roots and development of constructivist grounded theory. *Nurse Researcher* 2014;21(5):8–13.

21 Uganda Bureau of Statistics. Projected population by district (2015-2030) 2019.

22 Uganda Bureau of Statistics, UNICEF, World Bank. Poverty Maps of Uganda: Mapping the Spatial Distribution of Poor Households and Child Poverty Based on Data from the 2016/17 Uganda National Household Survey and the 2014 National Housing and Population Census. Technical Report 2019.

23 Strauss AL, Corbin JM. Basics of qualitative research: Techniques and procedures for developing grounded theory, 2nd edn. Thousand Oaks: Sage Publ 2003.

24 Taber K. Classroom-based research and evidence-based practice: Anintroduction. Los Angeles, Calif.: SAGE 2013.

25 Andersen R, Davidson P. Improving access to care in America: Individual and contextual indicators. *Changing the US Health Care System: Key Issues in Health Services Policy and Management* 2014;2007.

26 Biyik I, Usturali Mut AN, Albayrak M, et al. Effect of health literacy on help-seeking behavior: A comparison of patients accepting surgery and refusing surgery for urinary incontinence. *J Gynecol Obstet Hum Reprod* 2021;50(4):101908.

27 Roh YH, Lee BK, Park MH, et al. Effects of health literacy on treatment outcome and satisfaction in patients with mallet finger injury. *J Hand Ther* 2016;29(4):459–64. doi:10.1016/j.jht.2016.06.004 [published Online First: 17 October 2016].

28 Oliveira GS de, McCarthy RJ, Wolf MS, et al. The impact of health literacy in the care of surgical patients: a qualitative systematic review. *BMC Surg* 2015;15:86. doi:10.1186/s12893-015-0073-6 [published Online First: 17 July 2015].

29 Butler EK, Tran TM, Fuller AT, et al. Quantifying the pediatric surgical need in Uganda: results of a nationwide cross-sectional, household survey. *Pediatric surgery international* 2016;32(11):1075–85. doi:10.1007/s00383-016-3957-3 [published Online First: 12 September 2016].

30 Komenaka IK, Nodora JN, Machado L, et al. Health literacy assessment and patient satisfaction in surgical practice. *Surgery* 2014;155(3):374–83. doi:10.1016/j.surg.2013.10.011 [published Online First: 14 October 2013].

31 Fuller AT, Corley J, Tran TM, et al. Prevalence of Surgically Untreated Face, Head, and Neck Conditions in Uganda: A Cross-Sectional Nationwide Household Survey. *World neurosurgery* 2018;110:e747-e754. doi:10.1016/j.wneu.2017.11.099 [published Online First: 29 November 2017].

32 Davé DR, Nagarjan N, Canner JK, et al. Global Burden of Craniofacial Disorders: Where Should Volunteering Plastic Surgeons and Governments Focus Their Care? *J Craniofac Surg* 2020;31(1):121–24.

33 Raykar NP, Yorlets RR, Liu C, et al. The How Project: understanding contextual challenges to global surgical care provision in low-resource settings. *BMJ Global Health* 2016;1(4):e000075. doi:10.1136/bmjgh-2016-000075 [published Online First: 16 December 2016].

34 Close KL, Christie-de Jong FTE. Lasting impact: a qualitative study of perspectives on surgery by adult recipients of free mission-based surgical care in Benin. *BMJ Open* 2019;9(11):e028235. doi:10.1136/bmjopen-2018-028235 [published Online First: 7 November 2019].

For peer review only

35 White L. Speaking With Vampires: Rumor and History in Colonial Africa. Berkeley, Calif.: University of California Press 2000.
36 Scheper-Hughes N. Parts unknown. *Ethnography* 2004;5(1):29–73.
37 Bearden A, Fuller AT, Butler EK, et al. Rural and urban differences in treatment status among children with surgical conditions in Uganda. *PLoS One* 2018;13(11):e0205132.
38 Mwaka AD, Okello ES, Wabinga H. Perceptions and beliefs of lay people from northern Uganda regarding surgery for diagnosis and treatment of cervical cancer. *Psychooncology* 2018;27(8):1965–70.
39 Baine SO, Kasangaki A, Baine EMM. Task shifting in health service delivery from a decision and policy makers' perspective: a case of Uganda. *Hum Resour Health* 2018;16(1):1–20. doi:10.1186/s12960-018-0282-z [published Online First: 2 May 2018].
40 Bohren MA, Titiloye MA, Kyaddondo D, et al. Defining quality of care during childbirth from the perspectives of Nigerian and Ugandan women: A qualitative study. *Int J Gynaecol Obstet* 2017;139 Suppl 1:4–16. doi:10.1002/ijgo.12378 [published Online First: 7 December 2017].
41 World Health Organization. WHO traditional medicine strategy 2014 - 2023. Genève: WHO 2013.
42 Abdullahi AA. Trends and challenges of traditional medicine in Africa. *Afr J Tradit Complement Altern Med* 2011;8(5 Suppl):115–23. doi:10.4314/ajtcam.v8i5S.5 [published Online First: 3 July 2011].
43 Kagoya HR, Kibuule D, Mitonga-Kabwebwe H, et al. Awareness of, responsiveness to and practice of patients' rights at Uganda's national referral hospital. *Afr J Prim Health Care Fam Med* 2013;5(1).
44 Stephens T, Mezei A, O'Hara NN, et al. When Surgical Resources are Severely Constrained, Who Receives Care? Determinants of Access to Orthopaedic Trauma Surgery in Uganda. *World J Surg* 2017;41(6):1415–19. https://pubmed.ncbi.nlm.nih.gov/28097413/.
45 Mauti G, Githae M. Medical error reporting among physicians and nurses in Uganda. *Afr Health Sci* 2019;19(4):3107–17. https://pubmed.ncbi.nlm.nih.gov/32127887/.
46 Katongole Simon Peter, Robert Anguyo, Ddm Onzima, et al. Common Medical Errors and Error Reporting Systems in Selected Hospitals of Central Uganda 2015.
47 Kiguba R, Waako P, Ndagije HB, et al. Medication Error Disclosure and Attitudes to Reporting by Healthcare Professionals in a Sub-Saharan African Setting: A Survey in Uganda. *Drugs Real World Outcomes* 2015;2(3):273–87.
48 Barton SJ, Sandhu S, Doan I, et al. Perceived barriers and supports to accessing community-based services for Uganda's pediatric post-surgical population. *Disabil Rehabil* 2019:1–12.
49 Mangwi Ayiasi R, Kolsteren P, Batwala V, et al. Effect of Village Health Team Home Visits and Mobile Phone Consultations on Maternal and Newborn Care Practices in Masindi and Kiryandongo, Uganda: A Community-Intervention Trial. *PLoS One* 2016;11(4):e0153051. doi:10.1371/journal.pone.0153051 [published Online First: 21 April 2016].

Figure Legend

Figure 1. Sampling strategy per district. The goal was to conduct 6 interviews with FPs, 3 interiews with CMs from 3 different communities, 3 interviews with HWs (one each from a PFP, PNFP and public facility) and 1 interview with the DHO.

For peer review only

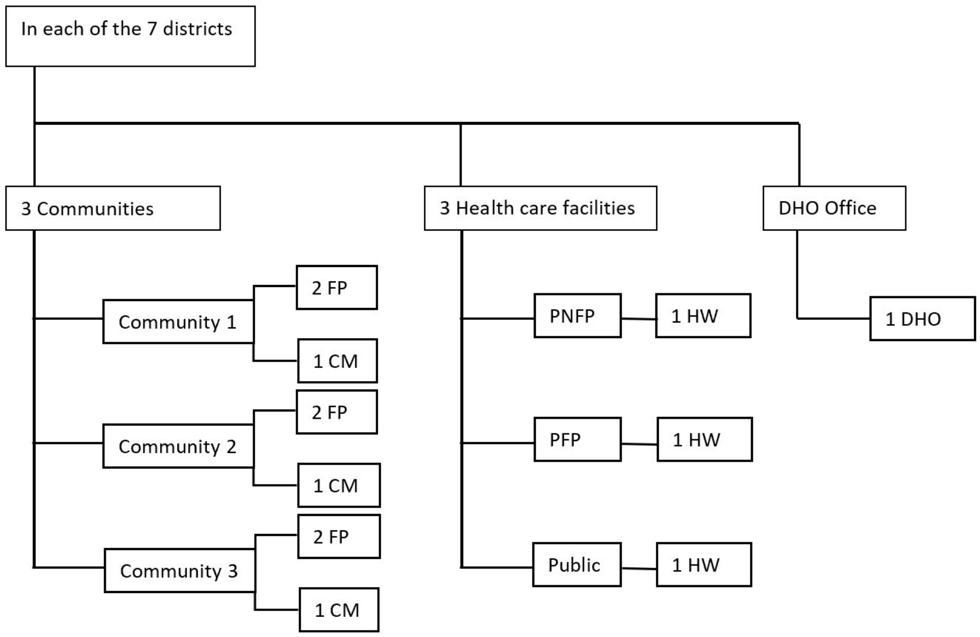

Sampling strategy per district. The goal was to conduct 6 interviews with FPs, 3 interviews with CMs from 3 different communities, 3 interviews with HWs (one each from a PFP, PNFP and public facility) and 1 interview with the DHO.

114x75mm (300 x 300 DPI)

Appendix 1 - Interview Guide District Health Officers

1. In your opinion, what are the main obstacles for the provision of access to healthcare?
2. In your opinion, what are the main obstacles to the provision of surgical care?
3. How do the supply chains for medical equipment work?
4. How do the supply chains for medication work?
5. Which interventions are planned in order to improve access to healthcare, especially to surgical care?
6. Are there campaigns or continuous educational programmes to inform the public about surgical conditions and access to surgical care? Have they proven effective?
7. How much importance is given to the provision of surgery in policy making?
8. What do you see as the most effective measure to increase the surgical workforce?
9. In your opinion, what would be the most effective way for NGOs involved in health care to support the local structures?
10. How could short stays of specialists, e.g. for eye or reconstructive surgery or fistula repair, be integrated into public surgical care for all?

For peer review only

Appendix 2 - Interview Guide Health professionals

Age:

Gender:

Profession:

Place of work:

Position:

Time period of work at current work place:

1. Please tell me about your personal experience with the provision of surgical care.
2. What are the key obstacles that [profession of interviewee] face at this health care facility?
3. What are the main difficulties in the provision of surgical care?
4. How do you deal with these difficulties?
5. How is the aftercare of surgical procedures organised and do you think it works effectively?
6. According to your experience with surgical care, what do you think are the main areas that need to be improved on?
7. What do you perceive as the main difficulties for patients?
8. In your opinion, what is the best way to address these difficulties for the patients?
9. Would you say patients trust in the expertise of health care professionals? How do patients perceive surgical conditions and treatment in your experience?
10. What are areas that you would describe as well-functioning when it comes to surgical care?

For peer review only

Appendix 3 - Interview Guide – Community Members (Former patients)

Age:

Education:

Occupation:

When were you operated?

Surgical condition:

Where were you operated from?

Length of hospital stay:

1. Please tell me about your experience with surgery.
2. How much time passed from your first symptoms to the diagnosis? And from the diagnosis to the surgical treatment?
3. Were you at any point unsure whether to have surgery and if yes why?
4. Did you consult any party outside of the hospital concerning the health condition you were in?
5. Who took the final decision of whether you would have the surgery?
6. Are you covered by a health insurance?
7. Were there any struggles to reach the hospital?
8. How did the health workers treat you? Were you respected? Were you taken care of?
9. How well did you feel informed about the procedure?
10. How successful was the operation in your opinion?
11. How was the aftercare of the operation? Where was it done? Did you face any struggles taking care of the wound after being discharged from hospital? Did you go through any episodes of extreme pain after the operation?
12. How did your operation change your attitude towards surgery? [This question was amended and changed to: If someone in your family needed to have surgery, what would you advise them?]
13. Throughout the entire process what were the main struggles you faced? In your opinion, which suggestions do you have to improve health facilities in Uganda or your community?
14. In which way did your community support you during that operation and your stay at the hospital?
15. Do you have any questions concerning what we have discussed with you?

For peer review only - http://bmjopen.bmj.com/site/about/guidelines.xhtml

Appendix 4 - Interview Guide – Community Members (general population)

Age:

Education:

Occupation:

1. Please tell me about your thoughts on surgery.
2. What do you think are the biggest barriers to surgical care that members of your community face?
3. Do you know where the closest surgical facility is and how long would it take you to get there?
4. What do you think are health problems for which surgery is needed?
5. Do you go to healthcare facilities regularly for check-up or only in case you experience symptoms?
6. How would you estimate the financial impact of a surgical operation on your livelihood?
7. Are you covered by a health insurance?
8. Would you be concerned if you were told by a healthcare provider that you had to have surgery?
9. If yes, what would be your major concerns?
10. Which things would you take into consideration before opting for surgery?
11. In your opinion, are patients treated right by healthworkers at the hospital?
12. How do people in this community support each other in case someone needs to go to the hospital?
13. Do you think consultations with traditional healers are helpful for possible surgical conditions?
14. Do you have traditional healers in your area? If yes, why do you think patients first consult them instead of going to the health facility directly? For the treatment of which cases are these traditional healers best known for?
15. Which measures would improve access to surgical care for your community in your opinion?

For peer review only - http://bmjopen.bmj.com/site/about/guidelines.xhtml

Standards for Reporting Qualitative Research (SRQR)*

http://www.equator-network.org/reporting-guidelines/srqr/

Title and abstract

	Page/line no(s).
**Title** - Concise description of the nature and topic of the study Identifying the study as qualitative or indicating the approach (e.g., ethnography, grounded theory) or data collection methods (e.g., interview, focus group) is recommended	3/3-5
**Abstract** - Summary of key elements of the study using the abstract format of the intended publication; typically includes background, purpose, methods, results, and conclusions	3/8-32

Introduction

**Problem formulation** - Description and significance of the problem/phenomenon studied; review of relevant theory and empirical work; problem statement	4/5-30
**Purpose or research question** - Purpose of the study and specific objectives or questions	4/32-38

Methods

**Qualitative approach and research paradigm** - Qualitative approach (e.g., ethnography, grounded theory, case study, phenomenology, narrative research) and guiding theory if appropriate; identifying the research paradigm (e.g., postpositivist, constructivist/ interpretivist) is also recommended; rationale**	4/43-49
**Researcher characteristics and reflexivity** - Researchers' characteristics that may influence the research, including personal attributes, qualifications/experience, relationship with participants, assumptions, and/or presuppositions; potential or actual interaction between researchers' characteristics and the research questions, approach, methods, results, and/or transferability	15/48-54
**Context** - Setting/site and salient contextual factors; rationale**	4/55-59
**Sampling strategy** - How and why research participants, documents, or events were selected; criteria for deciding when no further sampling was necessary (e.g., sampling saturation); rationale**	4/60-5/17
**Ethical issues pertaining to human subjects** - Documentation of approval by an appropriate ethics review board and participant consent, or explanation for lack thereof; other confidentiality and data security issues	15/14-19
**Data collection methods** - Types of data collected; details of data collection procedures including (as appropriate) start and stop dates of data collection and analysis, iterative process, triangulation of sources/methods, and modification of procedures in response to evolving study findings; rationale**	5/49-6/4

For peer review only - http://bmjopen.bmj.com/site/about/guidelines.xhtml

For peer review only

**Data collection instruments and technologies** - Description of instruments (e.g., interview guides, questionnaires) and devices (e.g., audio recorders) used for data collection; if/how the instrument(s) changed over the course of the study	5/23-45
**Units of study** - Number and relevant characteristics of participants, documents, or events included in the study; level of participation (could be reported in results)	6/19
**Data processing** - Methods for processing data prior to and during analysis, including transcription, data entry, data management and security, verification of data integrity, data coding, and anonymization/de-identification of excerpts	6/6-7
**Data analysis** - Process by which inferences, themes, etc., were identified and developed, including the researchers involved in data analysis; usually references a specific paradigm or approach; rationale**	6/11-16
**Techniques to enhance trustworthiness** - Techniques to enhance trustworthiness and credibility of data analysis (e.g., member checking, audit trail, triangulation); rationale**	

Results/findings

**Synthesis and interpretation** - Main findings (e.g., interpretations, inferences, and themes); might include development of a theory or model, or integration with prior research or theory	8/29-34
**Links to empirical data** - Evidence (e.g., quotes, field notes, text excerpts, photographs) to substantiate analytic findings	8/36-12/24

Discussion

**Integration with prior work, implications, transferability, and contribution(s) to the field -** Short summary of main findings; explanation of how findings and conclusions connect to, support, elaborate on, or challenge conclusions of earlier scholarship; discussion of scope of application/generalizability; identification of unique contribution(s) to scholarship in a discipline or field	12/28-14/32
**Limitations** - Trustworthiness and limitations of findings	14/35-57

Other

**Conflicts of interest** - Potential sources of influence or perceived influence on study conduct and conclusions; how these were managed	15/39-40
**Funding** - Sources of funding and other support; role of funders in data collection, interpretation, and reporting	15/44

*The authors created the SRQR by searching the literature to identify guidelines, reporting standards, and critical appraisal criteria for qualitative research; reviewing the reference lists of retrieved sources; and contacting experts to gain feedback. The SRQR aims to improve the transparency of all aspects of qualitative research by providing clear standards for reporting qualitative research.

For peer review only

**The rationale should briefly discuss the justification for choosing that theory, approach, method, or technique rather than other options available, the assumptions and limitations implicit in those choices, and how those choices influence study conclusions and transferability. As appropriate, the rationale for several items might be discussed together.

Reference:

O'Brien BC, Harris IB, Beckman TJ, Reed DA, Cook DA. **Standards for reporting qualitative research: a synthesis of recommendations.** *Academic Medicine*, Vol. 89, No. 9 / Sept 2014 DOI: 10.1097/ACM.0000000000000388

For peer review only

For peer review only - http://bmjopen.bmj.com/site/about/guidelines.xhtml
